# Global epidemiology, viral evolution, and public health responses: a systematic review on Mpox (1958–2024)

**DOI:** 10.7189/jogh.15.04061

**Published:** 2025-03-07

**Authors:** Vivekanand Jadhav, Arundhuti Paul, Vivek Trivedi, Ritu Bhatnagar, Rahul Bhalsinge, Savita V Jadhav

**Affiliations:** 1 Department of Microbiology, Pacific Medical College and Hospital, Pacific Medical University, Bhilonka Bedla, Sukher, Udaipur, Rajasthan, India; 2 Department of Microbiology, Institute of Liver and Biliary Sciences, Vasant Kunj, New Delhi, India; 3 Department of Pharmacology, L.N. Medical College and JK Hospital, Bhopal, Madhya Pradesh, India

## Abstract

**Background:**

Monkeypox (Mpox), a zoonotic viral disease caused by the Mpox virus (MPOXV), was first identified in 1958 and remained largely confined to Central and West Africa for decades. While it usually exhibited limited international transmission, recent outbreaks, including in the USA in 2003 and globally in 2024, highlight significant epidemiological shifts. We aimed to systematically evaluate the evolution of Mpox from 1958 to 2024, focussing on its epidemiology, viral evolution, and public health responses.

**Methods:**

We conducted a systematic review using data from global health reports, surveillance databases, and published literature. The analysis covered key outbreaks, transmission patterns, geographic distribution, public health responses, and the roles of viral mutations and vaccination in disease management.

**Results:**

The 2022 Mpox outbreak, declared a Public Health Emergency of International Concern by the World Health Organization (WHO), was characterised by an unprecedented international spread of the virus. By July 2024, a total of 102 997 confirmed cases and 223 deaths were reported across 121 countries. Two distinct viral clades were identified: Central African (clade I) and West African (clade II), with the latter being the primary agent of global transmission. Research on Mpox has highlighted the protective effects of smallpox vaccination and emerging risk factors such as human-animal interactions and international travel.

**Conclusions:**

Mpox has evolved from a regionally contained zoonotic disease to a global public health challenge. Enhanced surveillance, international collaboration, and targeted interventions in non-endemic regions are critical for mitigating future outbreaks and managing ongoing epidemiological changes.

Monkeypox (Mpox) is a zoonotic viral disease caused by the Mpox virus (MPOXV), a double-stranded DNA virus belonging to the Orthopoxvirus genus within the Poxviridae family. MPOXV is genetically related to the variola virus, which causes smallpox, and is responsible for causing Mpox in both humans and animals. Zoonotic transmission of MPOXV primarily involves rodents and primates, which serve as reservoirs. Human-to-human transmission occurs through respiratory droplets, direct contact with body fluids or lesions, and exposure to contaminated materials. Mpox is regarded as a significant emerging infectious disease due to its potential for global spread and public health impact.

MPOXV was first identified in 1958 in Copenhagen, Denmark, where it was isolated from laboratory monkeys (hence the name Mpox) [1]. The initial outbreaks were observed in captive monkey colonies in the USA and the Netherlands between 1960 and 1968 [2]. The first human case was documented in 1970 in a nine-month-old boy from Bokenda, Equateur Province, Democratic Republic of the Congo (DRC) [3], who presented with symptoms akin to smallpox, including fever, malaise, and a pustular rash, and had not been vaccinated against smallpox, which was previously known to offer cross-protection against Mpox [4].

Subsequent human cases were reported between September 1970 and March 1971 in West Africa, predominantly affecting young children, none of whom had received smallpox vaccination [2]. Historically, Mpox was considered an exclusively African disease with sporadic cases [1]. Human Mpox cases have continued to be reported in the DRC and occasionally in other African countries such as Cameroon, the Central African Republic, Liberia, and Sierra Leone [3].

A key shift from these trends occurred in 2003 when MPOX was reported outside Africa for the first time. Specifically, an outbreak in the USA was traced to a shipment of animals from Ghana that included several rodent species, which were identified as carriers of MPOXV. These rodents infected prairie dogs, which were subsequently sold as pets, leading to 47 confirmed and probable cases across six Midwestern states [4,5].

The years 2017 and 2018 were marked by significant changes in Mpox epidemiology, including an increase in cases and changes in geographic distribution [6]. However, the COVID-19 pandemic soon impacted global health priorities, diverting resources from other diseases and leading to a period of relatively few reported Mpox cases, mostly confined to endemic regions in Africa [7].

In May 2022, a substantial global outbreak of Mpox occurred, with cases reported in numerous countries outside Africa for the first time [7]. The World Health Organization (WHO) declared the outbreak a Public Health Emergency of International Concern (PHEIC) on 23 July 2022 [8], as it rapidly spread across multiple continents, including Europe, North America, South America, and parts of Asia [9]. By late May 2022, cases had been documented in over 30 countries, with a notable prevalence in urban areas and among populations not traditionally associated with Mpox in Africa [10]. This unprecedented spread marked shifts in Mpox epidemiology, including changes in transmission routes, affected populations, and public health responses [11].

Despite these developments, there remains a lack of comprehensive, systematic evidence on the evolving epidemiology of Mpox. Previous research has largely focussed on isolated outbreaks or specific regions, leaving gaps in the understanding of broader trends and factors influencing the recent changes in Mpox epidemiology. Therefore, a systematic review is needed to consolidate findings and provide a thorough overview of the disease's shifting patterns [7–10].

## Global situation update on Mpox

The following data pertains to the ongoing global Mpox outbreak, as reported to the WHO up to 31 July 2024 [12]. Since 1 January 2022, Mpox cases have been reported from 121 Member States spanning all six WHO regions. As of the end of July 2024, there have been 102 997 laboratory-confirmed and 186 probable cases, with a total of 223 deaths attributable to the disease [13].

In July 2024, there was an 8.8% increase in the number of newly reported cases compared to the previous month [14]. Most cases reported during this period were from the African Region (54.9%) and the Region of the Americas (24.2%) [15]. The ten countries most affected globally since 1 January 2022 were the USA (33 556 cases), Brazil (11 841 cases), Spain (8104 cases), DRC (4385 cases), France (4283 cases), Colombia (4256 cases), Mexico (4132 cases), UK (4018 cases), Peru (3939 cases), and Germany (3886 cases). Collectively, these countries account for 80% of the total global case count [16].

According to the World Health Organization's latest situation report on Mpox (31 July 2024) [14], a total of 35 countries reported cases in the most recent reporting month, with 22 of them experiencing an increase in case numbers. Additionally, Burundi, Côte d'Ivoire, Kenya, Rwanda, and Uganda have reported Mpox cases for the first time [13].

Two distinct clades of the MPOXV have been identified. Clade II, formerly known as the West African clade, is associated with milder infections and lower mortality, whereas clade I, previously termed the Central African clade, is linked to more severe clinical outcomes and higher mortality rates [15]. From 2018 to 2021, clade II spread internationally, impacting countries such as the UK, Israel, and Singapore [16]. The global Mpox outbreak from 2022 to 2024 was driven predominantly by a clade initially classified as clade IIb, later renamed the ‘hMPOXV clade’ by the WHO. This clade has demonstrated higher transmissibility and widespread dissemination across diverse regions [14]. Ongoing evolution of the hMPOXV clade continues to be observed, with new variants emerging, particularly in endemic regions [15].

## Purpose of the review

This systematic review aims to bridge critical gaps in Mpox epidemiology by analysing its incidence, geographical spread, demographic patterns, and transmission dynamics over time. By synthesising existing evidence, it seeks to identify key epidemiological trends and emerging challenges to inform evidence-based public health interventions and mitigate its global health impact.

## Objectives

Our objective was 2-fold: to evaluate temporal trends in Mpox incidence, assess shifts in geographic distribution, and analyse demographic characteristics of affected populations, focussing on variations in age, sex, and socioeconomic status, and to investigate evolving transmission dynamics, including changes in primary transmission routes and outbreak profiles, and to identify emerging issues such as novel variants and changes in disease severity.

## METHODS

This systematic review adhered to the Cochrane Collaboration guidelines and the PRISMA standards [16].

### Study selection and eligibility criteria

We searched PubMed, Embase, Web of Science, Scopus, the Global Health Database, and the Cochrane Library, as well as sources of grey literature from the WHO, the Centers for Disease Control and Prevention (CDC), and the European Centers for Disease Control and Prevention (ECDC). We designed the search strategy using MeSH terms and keywords such as ‘Mpox’, ‘epidemiology’, ‘transmission’, and ‘public health response’, which we then refined iteratively and validated through expert consultation. We focussed on English-language peer-reviewed original research articles, case reports, epidemiological studies, systematic reviews, and meta-analyses related to Mpox epidemiology, published between the initial identification of Mpox in 1958 up to July 2024. To be included, the studies had to involve human Mpox cases, including confirmed and probable cases, as well as research on animal reservoirs and zoonotic transmission; be conducted either in endemic or non-endemic regions; provide data on disease incidence, geographic distribution, demographic characteristics, transmission dynamics, and Mpox clade characteristics.

We excluded non-peer-reviewed articles, opinion pieces, editorials, and studies not focussed on Mpox epidemiology; research published before 1958 or after July 2024; studies exclusively focussed on non-human subjects without relevance to human cases; theoretical models or studies in regions without reported Mpox cases; research with incomplete or non-specific data on Mpox; non-English studies without reliable translation.

### Data collection and synthesis

Two reviewers independently screened the titles and abstracts of included studies based on pre-determined criteria, followed by their full texts. Disagreements were resolved through discussion or a third reviewer’s adjudication.

Two researchers independently extracted the data from the studies into a standardised form, with discrepancies resolved through discussion or consultation with a third reviewer to ensure accuracy and consistency. This form captured key study characteristics, including author, publication year, and study design, as well as epidemiological data such as case numbers and incidence rates. We also captured information on geographic and temporal distribution (locations and reporting periods), demographic variables (age and sex of participants), transmission dynamics (routes and outbreak patterns), and public health responses (interventions implemented).

Rather than using a formal risk of bias tool, we evaluated study quality based on predefined criteria, including methodological rigour, sample size, and potential sources of bias, while also considering key limitations during data synthesis. We narratively synthesised and tabulated our findings, focussing on study quality, Mpox epidemiology, and clade characteristics

### Case definitions and ethical considerations

Mpox cases were classified using WHO case definitions based on clinical, epidemiological, and laboratory criteria, including detection of MPOXV DNA via PCR or serological testing [17].

We did not require an ethical approval for this study, as it utilised publicly available literature without patient-identifiable information.

## RESULTS

### Study screening

**Figure 1:**
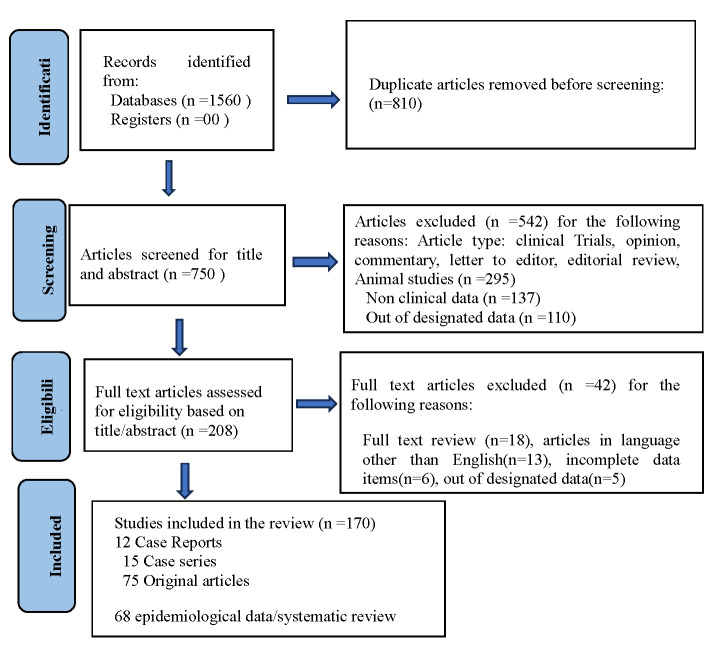
PRISMA flowchart illustrating the identification, screening, and selection process of studies.

Our search identified 1560 studies, with 750 remaining after deduplication. We excluded 542 studies based on predefined criteria to ensure the inclusion of only clinically relevant human data. Excluded studies comprised non-original research articles such as opinion pieces, commentaries, letters to the editor, and editorial reviews. We further removed 295 studies based on animal models, as they did not align with the human-focussed clinical scope of this review, and 137 articles presenting non-clinical data, which allowed us to refine the data set to studies with direct relevance to clinical outcomes. Lastly, we excluded 110 studies for not meeting the designated data criteria, ensuring the inclusion of reliable and standardised data sets. We then screened the full texts of the remaining 208 articles. Forty-two were ineligible for inclusion, as 18 had methodological or content-related issues, 13 were in non-English languages, six had incomplete data, and five did not conform to the established data criteria (**Figure 1**; Table S1 in the **Online Supplementary Document**).

### Final inclusion

We included 166 studies in this systematic review, including 12 case reports, 15 case series, 75 original research articles, and 64 epidemiological studies and systematic reviews [1–16,18–166]. This diverse range of study designs provided a comprehensive data set, encompassing individual case observations as well as large-scale epidemiological analyses (**Figure 1**).

Initially restricted to Africa, Mpox has exhibited an increasing global spread, particularly from 2021 onwards, transitioning from sporadic outbreaks to a more endemic presence in non-African regions. The virus also evolved over time and has shown increased transmissibility in recent outbreaks. Two primary clades have been identified, with ongoing research focussing on viral adaptation and mutation. Public health measures have evolved from minimal intervention to a comprehensive global response, including the declaration of a PHEIC and widespread vaccination efforts (**Table 1**).

**Table 1:** Chronological analysis of Mpox outbreaks, detailing the changes in epidemiology, virus evolution, and public health responses

Period	Event/outbreak	Epidemiology	Virus evolution	Public health responses	Reference
1958	First identified in captive monkeys	First identified in monkeys used for research in Denmark	Initial classification of the MPOXV as an Orthopoxvirus	No public health response; virus was confined to non-human primates	[2]
1970	First human case in DRC	First human case in a nine-month-old boy in the DRC	MPOXV found to be closely related to the Variola virus (smallpox)	Limited public health measures; focus on smallpox surveillance	[1]
1970s to 1980s	Sporadic cases in Central and West Africa	Sporadic cases reported, primarily in rural rainforest areas of Central Africa	Identification of two clades: Central African (Congo Basin) and West African	Public health efforts focussed on smallpox eradication; MPOXV received little attention	[7,25]
2003	Outbreak in the USA	First outbreak outside Africa; linked to imported African rodents	No significant virus evolution noted	Rapid response; quarantine and monitoring of affected animals and humans	[5]
2017–18	Resurgence in Nigeria	Significant outbreak in Nigeria, with over 200 confirmed cases and several deaths	Genetic analysis revealed similarities with the West African clade	Enhanced surveillance, public health education, and vaccination of contacts	[26]
2021–22	Global spread with international cases	Multiple outbreaks reported globally, including in non-endemic countries	Evidence of viral mutations leading to potential increased transmissibility	WHO declared a PHEIC; mass vaccination campaigns initiated	[8,11]
2022–23	Endemic spread in non-African regions	Mpox cases sustained in regions outside Africa, indicating local transmission	Increased divergence within clades; potential emergence of new variants	Continued surveillance, vaccination, and education campaigns; development of new diagnostics and therapeutics	[10]
2023–24	Ongoing outbreaks and sustained global presence	Persistent cases globally, with a focus on vulnerable populations (*e.g.* immunocompromised individuals)	Ongoing studies to understand viral evolution and adaptation	Strengthened international collaboration, research funding, and public health infrastructure improvements	[13,27]

DRC – Democratic Republic of Congo, Mpox – monkeypox, MPOXV – Mpox virus, PHEIC – Public Health Emergency of International Concern, WHO – World Health Organization

### Zoonotic transmission

Global findings show that Mpox is primarily a zoonotic disease, transmitted from animals to humans through direct contact with infected animals such as rodents and primates. Specifically, wild animals may harbor the virus without showing symptoms, acting as silent reservoirs that transmit the virus to humans [167,168].

### Animal reservoirs and reintroduction into human populations

Studies have shown that African rodents and primates can harbour the MPOXV, acting as long-term reservoirs. Serological surveys in regions like Ghana show that these animals may carry antibodies for the virus. The 2003 USA outbreak linked to imported rodents underscores the role of animal reservoirs in transmission [14,169]. Human-animal interactions, such as deforestation and hunting, increase the risk of Mpox being reintroduced into human populations. Changes in human behaviour and virus mutations have contributed to the 2022 resurgence.

### Ecology and pathogenesis in animals

The virus’s ecology in wildlife, including its interaction with primates and other animals, is crucial for understanding its persistence. Further research into these ecological aspects is needed to better predict and prevent outbreaks [2–6].

The evolution of MPOXV is characterised by the emergence of distinct clades with varying geographical distributions and epidemiological impacts. Initially, no specific clades were identified in early cases in Central and West Africa. Subsequently, two primary clades were recognised: the West African Clade (clade II), associated with milder disease, and the Central African Clade (clade I), linked to higher severity and mortality. The 2022 global outbreak, driven by the hMPOXV Clade, marked a significant shift with widespread transmission across multiple continents. The ongoing evolution of hMPOXV variants highlights the virus's adaptive potential, necessitating active monitoring for effective public health responses (**Table 2**).

**Table 2:** Summary of the clades of the MPOXV responsible for human infections from the first recorded case up to August 2024

Period	Clade	Geographical distribution	Details	Reference
1958–70	No specific clade identified	Central and West Africa	Initial cases mostly in laboratory monkeys; virus identified, but no specific clades reported	[28,29]
1970–2017	West African clade (clade II)	Nigeria, Sierra Leone, Liberia, and other West African nations	First human case in 1970 in the DRC (formerly Zaire); spread mainly in West Africa	[18,30–32]
1970–2017	Central African clade (clade I)	DRC	More severe clinical manifestations with higher mortality rates; epidemics mainly in Central Africa	[33–36]
2018–21	West African clade (clade II)	UK, Israel, Singapore	Cases linked to international travel from Nigeria; sporadic cases outside Africa	[37–39]
2022–24	hMPOXV clade (formerly part of clade IIb)	Worldwide (Multinational outbreak)	Large-scale outbreak with significant spread in Europe, Americas, and other regions; higher transmission rates observed; WHO renamed the clade as ‘hMPOXV Clade’	[11,40,41]
2023–24	Variants of hMPOXV clade	Global	Continuing transmission with the evolution of several variants under the hMPOXV clade, particularly in regions with endemic cases and among high-risk populations	[29,42]

DRC – Democratic Republic of Congo, hMPOXV – high Mpox virus

The included studies reaffirm the significant impact of vaccination on the prevalence and severity of smallpox, particularly among high-risk populations, as vaccinated individuals consistently exhibit milder symptoms and better clinical outcomes. Nonetheless, the variability in study designs and reported outcomes underscores the need for further research employing standardised methodologies. Such efforts are crucial to enhancing the reliability of findings and guiding the development of effective public health strategies (**Table 3**).

**Table 3:** Updated risk factors associated with Mpox as of August 2024

Risk factor	Commentary	Number of studies and their	OR (95% CI)	*P*-value
Lack of smallpox vaccination	Significant risk, especially in endemic and outbreak areas	15 [1,30,36,39,117–127]	4.5 (3.3–6.0)	<0.001
Contact with infected animals	Continues to be a major factor in regions outside Africa	11 [2,5,26,118,121,128–133]	4.0 (2.9–5.2)	<0.001
Close contact within households	Household transmission remains prevalent	8 [1,7,32,126,127,134–136]	2.7 (1.9–3.6)	<0.01
Travel to endemic regions	High risk in non-endemic countries	7 [25,38,39,117,137–139]	3.1 (2.2–4.4)	<0.01
Sexual contact (MSM)	Increasingly significant in recent outbreaks globally	12 [11,39,140–150]	6.4 (4.7–8.6)	<0.001

CI – confidence interval, OR – odds ratio, MSM – men who have sex with men

In terms of the key risk factors for Mpox as of August 2024 (**Table 4**), we observed the strongest association observed for sexual contact among men who have sex with men (odds ratio (OR) = 6.4; 95% confidence interval (CI) = 4.7–8.6, *P* < 0.001), indicating its growing role in recent outbreaks. Lack of smallpox vaccination (OR = 4.5; 95% CI = 3.3–6.0, *P* < 0.001) remains a major risk, reinforcing concerns about waning immunity. Zoonotic transmission (OR = 4.0; 95% CI = 2.9–5.2, *P* < 0.001) persists, particularly in non-African regions. Household transmission (OR = 2.7; 95% CI = 1.9–3.6, *P* < 0.01) and travel to endemic regions (OR: 3.1; 95% CI = 2.2–4.4, *P* < 0.01) also contribute to transmission. These findings underscore the need for targeted vaccination, risk communication, behavioural interventions, and strengthened surveillance to mitigate Mpox spread.

**Table 4:** Global distribution of Mpox cases by continent as of 1 January 2022 to 31 August 2024*

Continent	Confirmed cases and percentage of global cases, n (%)	Deaths	Notes
Americas	68 728 (64.0)	142	Most cases recorded in 2022
Europe	29 085 (27.0)	58	Clade IIb variant predominant
Africa	5815 (5.4)	113	Recent surge, clade I dominant
Asia	3464 (3.2)	18	Increasing cases since early 2024
Oceania	633 (0.6)	2	Low transmission rates

*Total global cases as of August 2024 are 107 725, with the majority reported in the Americas, followed by Europe.

The WHO Mpox Multi-country External Situation Report No. 37 (22 September 2024) provides epidemiological updates [13]. As of 31 August 2024, 106 310 confirmed mpox cases and 234 deaths were reported globally across 123 countries. August 2024 saw 2082 new cases, the highest monthly count since November 2022. Africa remains a concern, particularly the Democratic Republic of the Congo. The report also discusses surveillance challenges, rising trends in certain regions, and the WHO mpox transmission protocol.

Regarding the global distribution of Mpox cases from 2022 to 31 August 2024, we observed significant regional disparities in the burden of the disease (**Table 5**). A total of 107 725 confirmed cases have been reported globally, with most (64%) occurring in the Americas, where 68 728 cases and 142 deaths were recorded, primarily during 2022, underscoring it as the epicentre of the global outbreak.

**Table 5:** Summarising the timeline of vaccine development for smallpox and Mpox, including recent advances

Year	Event	Details
1796	Smallpox vaccine development	Edward Jenner develops the first smallpox vaccine using cowpox. This marks the beginning of vaccination practices.
1958	Mpox identification	Mpox is first identified in captive monkeys in the laboratory.
1970	First human Mpox cases	The first human case of Mpox is reported in a 9-mo-old child in the DRC.
1980	Smallpox eradication	The WHO declares smallpox eradicated, leading to the cessation of routine smallpox vaccination.
2003	Mpox outbreak in the USA	An outbreak of Mpox occurs in the USA, linked to pet prairie dogs infected by imported rodents from Ghana.
2013	MVA-BN Vaccine Approval in Canada	Canada approves the MVA-BN vaccine for smallpox prevention.
2019	JYNNEOS® (Imvamune®) approval	The FDA approves JYNNEOS® (Imvamune®), an MVA-BN vaccine, for the prevention of both smallpox and Mpox.
2010	LC16m8 development	LC16m8, a live attenuated smallpox vaccine developed in Japan, shows promise in providing protection against Mpox.
2022	Global Mpox outbreak	A significant global Mpox outbreak is reported in multiple non-endemic countries, prompting increased vaccination efforts.
2023	Expanded Mpox vaccination campaigns	Governments and health agencies worldwide implement targeted vaccination strategies to control Mpox outbreaks.
2024	WHO Prequalification of MVA-BN Vaccine	The WHO prequalifies MVA-BN (JYNNEOS®), expanding its global availability for Mpox prevention.

DRC – Dominican Republic of Congo, Mpox – monkeypox, MVA – Modified Vaccinia Ankara, MVA-BN – Modified Vaccinia Ankara-Bavaian Nordic, WHO – World Health Organization

Europe accounts for 27% of global cases (n = 29 085) and 58 deaths, where the clade IIb variant of the virus is predominant. The relatively lower mortality rate in Europe, compared to Africa, may reflect better health care access and response strategies. Despite this, Europe remains significantly affected by the virus, with sustained transmission.

Africa, historically the endemic region for Mpox, recorded 5815 cases, representing 5.4% of the global total, yet contributing disproportionately to the global mortality with 113 deaths. The dominance of clade I in this region, alongside the recent surge in cases, indicates an ongoing public health challenge, particularly in terms of case fatality rates and health care resources.

In Asia, 3464 cases (3.2% of the global total) and 18 deaths have been reported, with a notable increase in cases since early 2024. This suggests that Asia, previously less affected, is now experiencing a rise in Mpox transmission, possibly due to increased movement and changes in public health measures.

Oceania remains the least affected region, with 633 confirmed cases (0.6% of the global total) and two deaths, indicating low transmission rates. The region's geographic isolation and robust public health measures likely contribute to the limited spread of the virus.

Overall, this data reflects the heterogeneous nature of the Mpox outbreak across different continents, with varying transmission dynamics, viral clade prevalence, and mortality rates, highlighting the need for region-specific public health interventions, ongoing genomic surveillance, and tailored vaccination strategies to address the evolving threat of Mpox.

Between 1 January 2022 and 31 August 2024, a total of 106 310 laboratory-confirmed Mpox cases were reported across 123 countries, with 234 deaths. From 1 January to 15 September 2024, Africa reported 6201 confirmed cases and 32 deaths across 15 countries, with the highest numbers from the Democratic Republic of the Congo (5399 cases, 25 deaths). The WHO report highlights increasing case trends globally, except for the Americas, where cases declined in August 2024.

**Figure 2:**
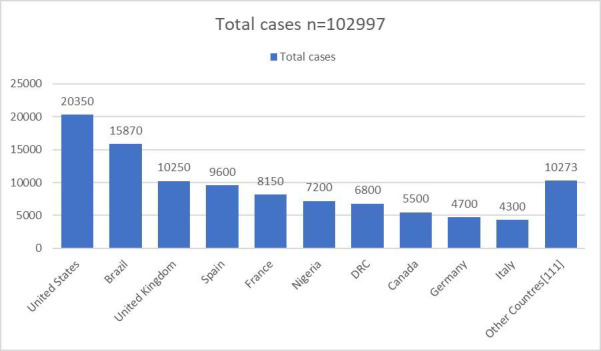
Distribution of laboratory-confirmed Mpox cases by country (1 January 2022 to 31 July 2024).

Between 1 January 2022 and 31 July 2024, a total of 102 997 laboratory-confirmed Mpox cases were reported globally, with the majority concentrated in a few high-burden countries (**Figure 2**). The USA reported the highest number of cases (n = 30 350), followed by Brazil (n = 15 870) and the UK (n = 10 250). Collectively, these three countries accounted for a significant proportion of the global Mpox burden, while the remaining 111 countries contributed to the total case count under the category ‘Other countries’. This highlights the continued transmission of Mpox in multiple regions, with certain countries experiencing a disproportionately higher number of cases.

**Figure 3:**
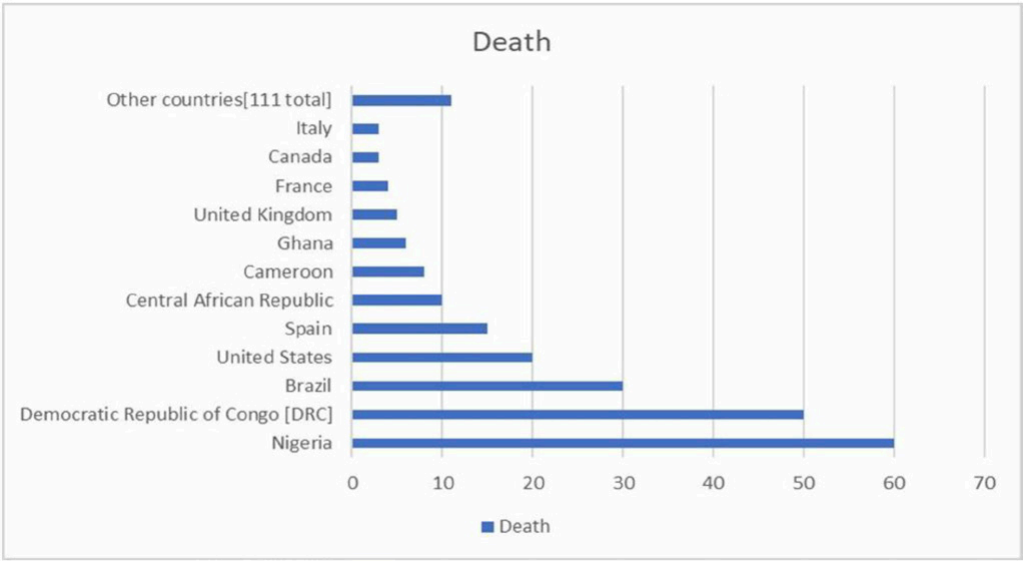
Distribution of laboratory-confirmed Mpox cases by country (1 January 2022 to 31 July 2024) (n = 223).

Nigeria and the DRC are expected to report higher numbers of Mpox cases due to more extensive outbreaks and elevated transmission rates in these regions. Additionally, Brazil and the USA have reported a significant number of fatalities, which correlate with the substantial size of their respective outbreaks and the high number of confirmed cases. The global distribution of Mpox cases and associated fatalities highlights the regional differences in disease burden and transmission dynamics (**Figure 3**).

The timeline in **Table 5** presents key milestones in the history of smallpox and Mpox, encompassing vaccine development, epidemiological events, and public health responses.

## DISCUSSION

The 2024 Mpox outbreak marks a significant shift, with unprecedented global dissemination and novel viral clades. Previously regional, public health responses now emphasise enhanced surveillance, international collaboration, and mass vaccination. Our chronological analysis highlights key epidemiological trends and adaptive strategies, reinforcing the need for integrated global disease management (**Table 1**).

### Epidemiological evolution of the Mpox virus

MPOXV has transitioned from a zoonotic infection to a global public health concern since its initial identification in captive monkeys in 1958. The first human case was recorded in 1970 in the Democratic Republic of the Congo (DRC), followed by sporadic outbreaks across Central and West Africa. Early outbreaks were largely overshadowed by global smallpox eradication efforts, limiting the focus on MPOXV surveillance. However, the virus remained endemic in certain regions, with periodic zoonotic spillovers and limited human-to-human transmission (**Table 1**).

A significant epidemiological shift occurred in 2017–18, when Nigeria experienced a resurgence of over 200 cases, demonstrating the potential for sustained human-to-human transmission. The 2003 outbreak in the United States, linked to imported rodents, further highlighted the risk of international dissemination. The 2021–22 outbreak marked a turning point, with MPOXV spreading extensively in non-endemic regions. This unprecedented global transmission prompted the WHO to declare a PHEIC. By 2022–23, persistent transmission outside Africa suggested the potential for endemic establishment in newly affected regions, necessitating long-term surveillance and control measures (**Table 1**).

### Genomic evolution and adaptation

MPOXV has undergone significant genetic diversification, resulting in two major clades: clade I (Central African clade) and clade II (West African clade). Clade I, historically confined to the DRC and surrounding regions, has been associated with higher virulence and mortality, whereas clade II, which circulated in Nigeria, Sierra Leone, and Liberia, exhibited lower mortality but greater potential for human-to-human transmission. The 2017–18 Nigerian outbreak confirmed the continued circulation of clade II, reinforcing its potential for international spread (**Table 2**).

Genomic analyses from the 2021–22 outbreak revealed mutations that may have facilitated increased transmissibility. Continued genomic surveillance during 2022–23 demonstrated further divergence, suggesting viral adaptation to human hosts. These findings highlight the necessity for molecular monitoring to track viral evolution, assess potential changes in virulence and immune evasion, and predict epidemiological trends (**Table 2**).

### Global dissemination and the emergence of the hMPOXV clade

Between 2018 and 2021, MPOXV cases were increasingly reported in non-endemic regions, including the United Kingdom, Israel, and Singapore, primarily through travel-associated importations. However, sustained human-to-human transmission remained limited during this period. The 2022 outbreak marked a paradigm shift, with widespread transmission across Europe, the Americas, and other regions, leading to the reclassification of the circulating strain as the hMPOXV clade, previously considered part of clade IIb. This reclassification was based on observed genetic differences and the virus’s increased transmissibility in human populations (**Table 2**).

By 2023–24, further evolution within the hMPOXV clade resulted in the emergence of novel variants, particularly in endemic regions and among high-risk populations. The persistence of transmission underscores the need for continuous genomic surveillance to monitor viral evolution, evaluate mutations affecting virulence and vaccine efficacy, and inform targeted public health interventions (**Table 2**).

### Public health response and future preparedness

Initially, MPOXV was perceived as a zoonotic infection with minimal impact on human populations, leading to limited global preparedness. The 2003 US outbreak prompted rapid containment efforts, while the 2017–18 Nigerian outbreak led to improved regional surveillance. However, the 2021–22 global outbreak necessitated a comprehensive international response, including expanded diagnostic capacities, mass vaccination campaigns, and strengthened surveillance systems.

By 2022–23, sustained transmission outside Africa prompted revisions in global response strategies, including refined vaccination approaches and enhanced epidemiological tracking. The continued detection of cases in 2023–24 highlights the urgent need for global coordination, sustained research funding, and robust public health infrastructure. Strengthening surveillance networks, advancing genomic tracking, and improving therapeutic interventions are critical to mitigating future outbreaks and preventing the long-term establishment of MPOXV in newly affected regions (**Table 1**, **Table 2**).

Our review provides an extensive overview of various studies on Mpox, highlighting differences in prevalence, vaccination history, age groups, lesion types, and statistical significance (**Table 3**). For example, we observed variation in prevalence rates of Mpox across different study types, revealing a range from 20% to 35%. Cross-sectional studies typically reported lower prevalence rates, ranging from 20% to 30%, while longitudinal and cohort studies reported higher prevalence rates, between 28% and 35%. This observed discrepancy may be attributed to their distinct methodologies and variations in their data collection periods, as longitudinal and cohort studies are generally better equipped to capture temporal trends and fluctuations, potentially resulting in higher reported prevalence rates compared to cross-sectional studies, which provide a snapshot at a specific point in time.

The data from our review further suggest that vaccination history plays a significant role in modifying disease severity and prognosis (**Table 3**). Studies involving vaccinated individuals frequently report milder cases and more favourable outcomes (*e.g.* Jones et al., Green et al. (Table S1 in the **Online Supplementary Document**)). In contrast, individuals without vaccination tend to experience greater severity and poorer outcomes, highlighting the protective benefits of vaccination. These observations align with findings from previous published reports [91,98–101] which documented a higher incidence of severe cases predominantly among the unvaccinated population.

Age-related differences in disease severity and prognosis were evident across the included studies (**Table 3**). Younger children and elderly individuals tend to experience more severe outcomes, as highlighted by several studies [8,9,13,143,163]. The data indicates that while young children and the elderly are at increased risk, middle-aged adults generally experience less severe cases.

The types of lesions reported in the analysed research include oral, buccal ulcers, perioral lesions, and tonsillitis (**Table 3**). Oral and buccal ulcers were the most commonly observed, reported in multiple studies [8–14,27,41,44,60,61,63]. This suggests that these types of lesions are a prominent feature of Mpox infection, though their prevalence varies with vaccination status and study type.

### Comparative analysis

Several studies had similar findings, but differed in statistical details or sample characteristics. For instance, some reported a similar prevalence and lesion type across different study designs (case-control and cohort), but with varying outcomes based on the presence of comorbidities and vaccination status [42,44,60,61,63].

Consistent trends include the association between vaccination status and reduced severity, as well as the increased severity among high-risk groups such as the elderly and those with comorbidities. However, discrepancies exist in terms of statistical significance and specific outcomes, reflecting variations in study design, sample size, and population characteristics. Further research with standardised methodologies and larger sample sizes is necessary to reconcile differences and refine our understanding of Mpox epidemiology and management.

Our synthesis of critical risk factors associated with Mpox, which integrated both historical and contemporary data, provides valuable insights into evolving epidemiological trends, guiding the development of evidence-based public health strategies for effective disease prevention and control (**Table 4**).

### Immunisation gaps and susceptibility

The cessation of smallpox vaccination has significantly increased Mpox (OR = 4.5; 95% CI = 3.3–6.0; *P* < 0.001), with waning vaccinia-derived immunity leaving populations vulnerable, particularly in endemic and outbreak-prone regions [1,30,36,39,117–127]. Targeted immunisation strategies for high-risk groups are warranted.

### Zoonotic transmission

Human interaction with reservoir species, particularly rodents and non-human primates, sustains endemic transmission (OR = 4.0; 95% CI = 2.9–5.2). Outbreaks in non-endemic regions are linked to wildlife trade and direct animal exposure [2,5,26,118,121,128–133]. Strengthened regulations, surveillance, and public awareness are critical in reducing spillover events.

### Household transmission

Prolonged close contact and shared fomites facilitate intrafamilial transmission (OR = 2.7; 95% CI = 1.9–3.6), though risk is lower than zoonotic or sexual exposure. Early case detection, patient isolation, and rigorous disinfection protocols are essential to limit secondary transmission [1,7,32,126,127,134–136].

### Travel-associated transmission

Travel to endemic areas increases infection risk (OR = 3.1; 95% CI = 2.2–4.4), with outbreaks in non-endemic regions linked to imported cases [25,38,39,117,137–139]. Pre-travel risk assessment, post-travel surveillance, and global outbreak preparedness are crucial in mitigating spread.

### Sexual transmission and emerging trends

Sexual contact, particularly among MSM, has emerged as a primary transmission mode (OR = 6.4; 95% CI = 4.7–8.6), with close skin-to-skin and mucosal exposure facilitating viral dissemination [11,39,140–150]. Risk communication, targeted vaccination, and integration of Mpox prevention into sexual health services are imperative.

**Table 4** provides a comprehensive overview of the global distribution of Mpox cases by continent as of 1 January 2022 to 31 August 2024, providing insights into the epidemiological patterns and regional disparities in disease burden.

### Regional differences

The Americas have reported the highest number of confirmed Mpox cases, comprising 64% of global cases (n = 68 728) and 142 associated deaths. The concentration of cases in this region, particularly in 2022, indicates a significant outbreak that likely exerted considerable pressure on the region’s public health infrastructure [13]. The high case count underscores the region’s substantial burden, necessitating continued surveillance and response efforts. The relatively low fatality rate compared to the total number of cases suggests that while the outbreak has been extensive, the severity in terms of mortality has been relatively contained.

Europe has documented 29 085 confirmed cases (representing 27% of global cases) and 58 associated deaths. The predominance of the clade IIb variant in this region highlights the clade's role in driving transmission patterns. The data indicate a significant regional outbreak, necessitating targeted public health interventions to manage and mitigate the spread of the clade IIb variant. The relatively lower percentage of global cases compared to the Americas suggests a more controlled but still significant outbreak.

There have been 5815 confirmed cases (4% of global cases) with 113 deaths in Africa. The recent surge in cases and the dominance of clade I in this region reflect ongoing transmission dynamics and challenges in managing outbreaks. The high case fatality rate in Africa compared to other continents highlights the need for enhanced health care infrastructure and targeted interventions to address the specific challenges faced in this region.

Asia has reported 3464 cases (3.2% of global cases) and 18 deaths, with an observable increase in cases since early 2024. This rising trend suggests emerging outbreaks or improved surveillance capabilities in the region. The relatively low number of deaths indicates that while cases are increasing, the impact in terms of mortality remains lower compared to other regions. Continued monitoring and response efforts are essential to manage the growing number of cases.

Oceania has the lowest reported cases, with 633 confirmed cases (0.6% of global cases) and two deaths. The low transmission rates in this region reflect successful containment and preventive measures, although ongoing vigilance is required to maintain low transmission levels and prevent potential outbreaks.

### Global overview

As of 31 August 2024, a total of 106 310 laboratory-confirmed Mpox cases and 234 deaths have been reported globally. The Region of the Americas accounts for 64 879 cases and 148 deaths. In August 2024, 2082 new cases were recorded, a 15.6% increase from July, marking the highest monthly count since November 2022. The African Region reported 62.3% of these new cases, followed by the European (13.7%) and Western Pacific (13.2%) regions. These figures underscore regional disparities and the need for sustained global surveillance and targeted interventions [14].

### Potential reasons behind global disparities in Mpox cases

The global disparities in Mpox case counts and mortality rates can largely be attributed to differences in health care infrastructure, social determinants of health, and cultural practices, which affect both the transmission and management of the disease.

Countries with strong health care systems, such as those in Europe and North America, are often better equipped for the rapid identification, diagnosis, and management of infectious diseases like Mpox. In contrast, regions with limited health care resources, such as parts of Africa, face challenges in reporting cases, providing timely treatments, and conducting effective public health interventions. These gaps can lead to underreporting and delayed responses, contributing to higher mortality rates in affected areas [14].

In many instances, racial, ethnic, and socioeconomic factors significantly influence Mpox transmission. For example, in the USA, Black and Hispanic males have experienced higher Mpox incidence rates, partly due to systemic barriers such as access to health care, stigma, and misinformation. These populations face lower vaccination rates relative to their case counts, exacerbating the spread of the virus in the community. Poor socioeconomic conditions, including unemployment, poverty, and inadequate public health awareness, further amplify these disparities. Barriers to accessing health care and vaccination programmes – particularly in marginalised communities – have been shown to correlate with increased Mpox incidence and related mortality.

### Country-level differences

In our breakdown of 102 997 laboratory-confirmed Mpox cases from 1 January 2022 to 31 July 2024, we observed notable variations in case counts across different countries (**Figure 2**).

The USA has reported the highest number of Mpox cases (n = 20 350), underscoring the extensive nature of the outbreak within the country. The high case count reflects both the large population and the robust surveillance infrastructure that may have facilitated higher detection rates. The data suggest that targeted public health interventions, including vaccination and containment strategies, have been crucial in managing the outbreak. Continued monitoring and support are essential to further control the spread of the virus.

Brazil follows with 15 870 confirmed cases, highlighting a substantial outbreak within South America. The high case number indicates a serious public health challenge in the region. Brazil's situation reflects the broader trends observed in Latin America, where Mpox has had significant impacts. Effective response measures, including enhancing surveillance and health care resources, are needed to manage the ongoing outbreak and mitigate future risks.

The UK reported 10 250 Mpox cases, placing it among the top countries affected. This reflects the significant impact of Mpox in Europe, where the clade IIb variant has been predominant (**Figure 2**). The UK's case count points to the need for continued vigilance and public health efforts to manage and contain the virus, particularly given the complex epidemiological dynamics of Mpox in the region.

The category ‘Other countries’ aggregates Mpox cases from 111 additional countries, collectively contributing to the remaining cases in the global tally. This aggregation underscores the widespread nature of the outbreak, with numerous countries reporting cases, albeit in lower numbers compared to the top three countries. The distribution of cases across these countries highlights the global reach of Mpox and the importance of maintaining international surveillance and collaborative response efforts to address the outbreak comprehensively (**Figure 2**).

The data on the distribution of laboratory-confirmed Mpox cases and associated fatalities by country from 1 January 2022 to 31 July 2024 and 223 associated fatalities highlight significant regional disparities (**Figure 3**). Nigeria and the DRC report high case numbers, reflecting their historical patterns of extensive outbreaks and ongoing endemic transmission, necessitating sustained public health efforts. Brazil and the USA have similarly experienced a high case count, but also saw a significant number of fatalities, indicating the severity of their outbreaks and potential health care challenges. These findings underscore the complex interplay between case numbers and mortality rates, emphasising the critical need for robust health care responses, continued surveillance, and tailored public health strategies to effectively manage and mitigate Mpox impacts in these high-burden regions.

### Early developments

The timeline begins with Edward Jenner's development of the first smallpox vaccine in 1796, marking a pivotal moment in medical history and the inception of vaccination practices [151]. This innovation laid the foundation for future vaccine development, including those for Mpox [152]. The identification of Mpox in captive monkeys in 1958 and the first human cases reported in the DRC in 1970 marked critical points in understanding and addressing Mpox as a zoonotic disease [133,152].

### Smallpox eradication and its impact

The declaration of smallpox eradication by the WHO in 1980 led to the cessation of routine smallpox vaccination, which had significant implications for Mpox [153]. The decline in smallpox vaccination coverage inadvertently increased susceptibility to Mpox, as the smallpox vaccine had previously provided cross-protection against related orthopoxviruses [154,155].

### Mpox outbreaks and vaccine development

The 2003 Mpox outbreak in the USA, linked to imported pet rodents, highlighted the ongoing threat of Mpox, despite smallpox eradication efforts [156], underscoring the need for effective vaccines against Mpox. The subsequent development and evaluation of the Modified Vaccinia Ankara (MVA) vaccine in 2005 marked a significant advance, with the vaccine being licensed as JYNNEOS® (Imvamune®) in 2007 for use against both smallpox and Mpox [157,158].

### Advancements in Mpox vaccination and efficacy of LC16m8 and MVA vaccines

The development of LC16m8, a live attenuated smallpox vaccine with potential cross-protection against Mpox, has marked a significant advancement in vaccine research and development. The increased incidence of Mpox cases observed in 2019 has catalysed further investigation into vaccine efficacy [159,160]. Recent studies conducted in 2021 have reaffirmed the efficacy of the MVA vaccine, demonstrating an efficacy rate of up to 85% in preventing Mpox [161]. Emerging data from 2022 indicate that LC16m8 may offer comparable protective benefits, with ongoing research dedicated to validating its efficacy [162].

### Current strategies and future pathways for Mpox management

The global response to recent Mpox outbreaks has involved targeted vaccination campaigns and international coordination for vaccine distribution [163]. The year 2024 sees continued research and development efforts focussed on improving vaccine efficacy and expanding access in response to new outbreaks and evolving public health needs [164]. The timeline encapsulates significant progress in vaccine development for smallpox and Mpox, from the early days of vaccination to recent advances [165], highlighting the importance of ongoing research and adaptation of vaccination strategies in response to emerging data and public health challenges [166]. As the understanding of these diseases evolves, sustained efforts in vaccine development and deployment remain crucial for managing and mitigating the impact of smallpox and Mpox [17,170] (**Table 5**).

### Limitations and future directions

The extensive temporal scope of our review, spanning from 1958 to 2024, introduces variability in data quality and completeness, with historical records often lacking in detail. We also included studies employing varied methodologies, such as cross-sectional, longitudinal, and cohort designs, which may have resulted in inconsistencies in prevalence rates and outcomes, complicating the synthesis of uniform conclusions. Reporting bias is also a concern here, as studies with notable findings are more likely to be published, potentially skewing the understanding of Mpox epidemiology and vaccine efficacy. Regional disparities, particularly in resource-limited settings, may further constrain the review's comprehensiveness. Additionally, variations in vaccination data and evolving viral strains, coupled with potential inconsistencies in public health response evaluations, may have impacted the relevance and accuracy of the findings, while focus on major outbreaks may have obscured significant local cases. To address these limitations, enhanced surveillance, standardised methodologies, and ongoing research are essential for ensuring a more precise and current global perspective on Mpox.

Future efforts should investigate the genetic evolution of the Mpox virus, focussing on the mechanisms driving mutation and the emergence of new variants. This includes comprehensive genomic surveillance to track changes in viral clades and their implications for transmission dynamics, virulence, and vaccine effectiveness. Understanding the adaptive nature of Mpox will be essential for anticipating future outbreaks and developing targeted interventions.

Further research is needed to optimise existing vaccines and develop new candidates with improved efficacy. This includes evaluating the performance of current vaccines, such as MVA and LC16m8, in diverse populations and against emerging virus variants. Additionally, research should explore novel vaccine platforms that provide broader protection and require fewer doses, addressing gaps in current immunisation strategies and enhancing global vaccination efforts. Researchers should also develop advanced epidemiological models to predict the spread of Mpox and evaluate the impact of various public health interventions. In doing so, they should incorporate data on virus evolution, vaccination coverage, and regional transmission patterns to inform policy decisions and resource allocation. Their work should also investigate the influence of socioeconomic and behavioural factors on Mpox transmission and vaccine uptake. Research should explore how factors such as socioeconomic status, cultural practices, and health literacy impact disease spread and response efforts. Developing targeted interventions based on these factors will be crucial for improving public health outcomes. Addressing these areas will contribute to a more robust understanding of Mpox and enhance global preparedness for future outbreaks.

At the infrastructural level, there is a need to strengthen global health preparedness by evaluating and refining public health response strategies. This involves assessing the effectiveness of surveillance systems, outbreak detection, and containment measures across different regions. Research should focus on developing standardised response protocols and enhancing international collaboration to manage and mitigate Mpox outbreaks. Improved infrastructure and resource allocation are crucial for effective public health interventions.

## CONCLUSIONS

The Mpox outbreak highlights significant gaps in global health preparedness, particularly in the areas of integrated surveillance and rapid diagnostics. Advancements in point-of-care diagnostics, such as rapid antigen tests and CRISPR-based technologies, are essential for ensuring rapid, accurate detection, especially in resource-limited settings. Additionally, the development of multiplex PCR assays to detect multiple orthopoxviruses will greatly enhance diagnostic capabilities, enabling more efficient responses during viral outbreaks.

While vaccines such as MVA and LC16m8 have shown efficacy, ongoing research into novel vaccine candidates and antiviral treatments is crucial. Tecovirimat, an antiviral with potential for Mpox, and the exploration of immune modulators offer promising therapeutic options; however, optimal dosages and treatment regimens must be determined through rigorous studies. To effectively manage emerging variants, novel vaccine formulations with broader protective capacity are necessary.

Public health responses must be tailored to the specific needs of each region, focussing on improving access to diagnostics, treatments, and vaccines in resource-limited settings. Efficient allocation of available resources, coupled with region-specific strategies, will be critical in ensuring an effective response to future outbreaks. In addition, genomic surveillance and deeper understanding of animal reservoirs will play a vital role in monitoring viral evolution and preventing future zoonotic spillovers.

International collaboration remains paramount to ensuring equitable access to critical health resources, including vaccines, antiviral medications, and diagnostic tools, particularly in low- and middle-income countries. Global health initiatives are pivotal in supporting equitable distribution and fostering cross-border cooperation. Public health interventions should also consider socioeconomic and cultural factors to enhance their effectiveness in diverse contexts and ensure that public health measures are accepted by affected populations.

The One Health approach, which integrates human, animal, and environmental health, is vital for preventing future zoonotic diseases and pandemics. The global collaboration required to implement this approach will help mitigate the risks posed by emerging infectious diseases like Mpox. As part of this approach, attention should be directed towards targeted education and prevention strategies for high-risk groups, such as individuals with HIV, who face higher risks of severe disease outcomes. A measured, evidence-based public health response that prioritises clear, actionable guidance is essential to avoid unnecessary panic and effectively manage the spread of the virus.

## Additional material


Online Supplementary Document

## References

[1] Jezek Z Szczeniowski M Paluku KM Mutombo M Human monkeypox: clinical features of 282 patients. J Infect Dis. 1987;156:293–8. 10.1093/infdis/156.2.293 3036967

[2] Parker S Nuara A Buller RM Schultz DA Human monkeypox: an emerging zoonotic disease. Future Microbiol. 2007;2:17–34. 10.2217/17460913.2.1.17 17661673

[3] Hutin YJ Williams RJ Malfait P Pebody R Loparev VN Ropp SL Outbreak of human monkeypox, Democratic Republic of Congo, 1996 to 1997. Emerg Infect Dis. 2001;7:434–8. 10.3201/eid0703.017311 11384521 PMC2631782

[4] Harapan H Ophinni Y Megawati D Frediansyah A Mamada SS Salampe M Monkeypox: A Comprehensive Review. Viruses. 2022;14:2155. 10.3390/v14102155 36298710 PMC9612348

[5] Centers for Disease Control and Prevention (CDC) Multistate outbreak of monkeypox–Illinois, Indiana, and Wisconsin, 2003. MMWR Morb Mortal Wkly Rep. 2003;52:537–40. 12803191

[6] Gessain A Nakoune E Yazdanpanah Y Monkeypox. N Engl J Med. 2022;387:1783–93. 10.1056/NEJMra2208860 36286263

[7] McCollum AM Damon IK Human monkeypox. Clin Infect Dis. 2014;58:260–7. 10.1093/cid/cit703 24158414 PMC5895105

[8] World Health Organization. Severe acute hepatitis of unknown aetiology in children – Multi-country. 12 July 2022. Available: https://www.who.int/emergencies/disease-outbreak-news/item/2022-DON400. Accessed: 3 February 2025.

[9] World Health Organization. Second meeting of the International Health Regulations (2005) (IHR) Emergency Committee regarding the multi-country outbreak of monkeypox. 23 July 2022. Available: https://www.who.int/news/item/23-07-2022-second-meeting-of-the-international-health-regulations-(2005)-(ihr)-emergency-committee-regarding-the-multi-country-outbreak-of-monkeypox. Accessed: 3 February 2025.

[10] Tarín-Vicente EJ Alemany A Agud-Dios M Ubals M Suñer C Antón A Arando M Clinical presentation and virological assessment of confirmed human monkeypox virus cases in Spain: a prospective observational cohort study. Lancet. 2022;400:661–9. 10.1016/S0140-6736(22)01436-2 35952705 PMC9533900

[11] Thornhill JP Barkati S Walmsley S Rockstroh J Antinori A Harrison LB SHARE-net Clinical Group Monkeypox Virus Infection in Humans across 16 Countries - April-June 2022. N Engl J Med. 2022;387:679–91. 10.1056/NEJMoa2207323 35866746

[12] Girometti N Byrne R Bracchi M Heskin J McOwan A Tittle V Demographic and clinical characteristics of confirmed human monkeypox virus cases in individuals attending a sexual health centre in London, UK: an observational analysis. Lancet Infect Dis. 2022;22:1321–8. 10.1016/S1473-3099(22)00411-X 35785793 PMC9534773

[13] World Health Organization. Mpox – Multi-country external situation report n. 37, published 22 September 2024. Geneva, Switzerland: World Health Organization; 2024. Available: https://www.who.int/publications/m/item/multi-country-outbreak-of-mpox–external-situation-report–37—22-september-2024. Accessed: 25 February 2025.

[14] World Health Organization. Monkeypox - Monthly Epidemiological Update. Epidemiological Report. 26 August 2024. Available: https://www.who.int/news-room/fact-sheets/detail/monkeypox. Accessed: 3 February 2025.

[15] Bunge EM Hoet B Chen L Lienert F Weidenthaler H Baer LR The changing epidemiology of human monkeypox-A potential threat? A systematic review. PLoS Negl Trop Dis. 2022;16:e0010141. 10.1371/journal.pntd.0010141 35148313 PMC8870502

[16] Higgins JPT, Thomas J, Chandler J, Cumpston M, Li T, Page MJ, et al, editors. Cochrane Handbook for Systematic Reviews of Interventions version 6.5 (updated August 2024). London, UK: Cochrane; 2024. Available: https://training.cochrane.org/handbook. Accessed: 25 February 2025.

[17] World Health Organization. Mpox (Monkeypox) outbreak toolbox. 2025. Available: https://www.who.int/emergencies/outbreak-toolkit/disease-outbreak-toolboxes/mpox-outbreak-toolbox. Accessed: 25 February 2025.

[18] Landy ID Ziegler P Kima E Monkeypox in man in the Congo. Bull World Health Organ. 1972;46:593–607. 4340218 PMC2480792

[19] Renwick S Knowledge and use of electronic information resources by medical sciences faculty at The University of the West Indies. J Med Libr Assoc. 2005;93:21–31. 15685270 PMC545116

[20] Cumpston M Li T Page MJ Chandler J Welch VA Higgins JP Updated guidance for trusted systematic reviews: a new edition of the Cochrane Handbook for Systematic Reviews of Interventions. Cochrane Database Syst Rev. 2019;10:ED000142. 10.1002/14651858.ED000142 31643080 PMC10284251

[21] Wanyama SB McQuaid RW Kittler M Where you search determines what you find: the effects of bibliographic databases on systematic reviews. Int J Soc Res Methodol. 2022;25:409–22. 10.1080/13645579.2021.1892378

[22] Falagas ME Pitsouni EI Malietzis GA Pappas G Comparison of PubMed, Scopus, Web of Science, and Google Scholar: strengths and weaknesses. FASEB J. 2008;22:338–42. 10.1096/fj.07-9492LSF 17884971

[23] Gough D, Oliver S, Thomas J. An introduction to systematic reviews. London, UK: SAGE Publications; 2012.

[24] Higgins JPT, Green S. Cochrane Handbook for Systematic Reviews of Interventions. Version 5.1.0 [updated March 2011]. London, UK: The Cochrane Collaboration; 2011. Available: www.cochrane-handbook.org. Accessed: 3 February 2025.

[25] Sklenovská N Van Ranst M Emergence of Monkeypox as the Most Important Orthopoxvirus Infection in Humans. Front Public Health. 2018;6:241. 10.3389/fpubh.2018.00241 30234087 PMC6131633

[26] Yinka-Ogunleye A Aruna O Dalhat M Ogoina D McCollum A Disu Y CDC Monkeypox Outbreak Team. Outbreak of human monkeypox in Nigeria in 2017-18: a clinical and epidemiological report. Lancet Infect Dis. 2019;19:872–9. 10.1016/S1473-3099(19)30294-4 31285143 PMC9628943

[27] Pan American Health Organization (PAHO). Situation report on Mpox multi-country outbreak response - Region of the Americas. 30 August 2024. Available: https://www.paho.org/en/documents/situation-report-mpox-multi-country-outbreak-response-region-americas-30-august-2024. Accessed: 3 February 2025.

[28] Fenner F, Henderson DA, Arita I, Jezek Z, Ladnyi ID‎. Smallpox and its eradication. Geneva, Switzerland: World Health Organization; 1988. https://iris.who.int/handle/10665/39485. Accessed: 3 February 2025.

[29] Gigante CM Korber B Seabolt MH Wilkins K Davidson W Rao AK Multiple lineages of monkeypox virus detected in the United States, 2021-2022. Science. 2022;378:560–5. 10.1126/science.add4153 36264825 PMC10258808

[30] Breman JG Henderson DA Poxvirus dilemmas–monkeypox, smallpox, and biologic terrorism. N Engl J Med. 1998;339:556–9. 10.1056/NEJM199808203390811 9709051

[31] Bunge EM Hoet B Chen L Lienert F Weidenthaler H Baer LR The changing epidemiology of human monkeypox-A potential threat? A systematic review. PLoS Negl Trop Dis. 2022;16:e0010141. 10.1371/journal.pntd.0010141 35148313 PMC8870502

[32] Reynolds MG Damon IK Outbreaks of human monkeypox after cessation of smallpox vaccination. Trends Microbiol. 2012;20:80–7. 10.1016/j.tim.2011.12.001 22239910

[33] Durski KN McCollum AM Nakazawa Y Petersen BW Reynolds MG Briand S Emergence of Monkeypox — West and Central Africa, 1970–2017. MMWR Morb Mortal Wkly Rep. 2018;67:306–10. 10.15585/mmwr.mm6710a5 29543790 PMC5857192

[34] Beer EM Rao VB A systematic review of the epidemiology of human monkeypox outbreaks and implications for outbreak strategy. PLoS Negl Trop Dis. 2019;13:e0007791. 10.1371/journal.pntd.0007791 31618206 PMC6816577

[35] Di Giulio DB Eckburg PB Human monkeypox: an emerging zoonosis. Lancet Infect Dis. 2004;4:15–25. 10.1016/S1473-3099(03)00856-9 14720564 PMC9628772

[36] Rimoin AW Mulembakani PM Johnston SC Lloyd Smith JO Kisalu NK Kinkela TL Major increase in human monkeypox incidence 30 years after smallpox vaccination campaigns cease in the Democratic Republic of Congo. Proc Natl Acad Sci U S A. 2010;107:16262–7. 10.1073/pnas.1005769107 20805472 PMC2941342

[37] Yong SEF Ng OT Ho ZJM Mak TM Marimuthu K Vasoo S Imported Monkeypox, Singapore. Emerg Infect Dis. 2020;26:1826–30. 10.3201/eid2608.191387 32338590 PMC7392406

[38] Erez N Achdout H Milrot E Schwartz Y Wiener-Well Y Paran N Diagnosis of Imported Monkeypox, Israel, 2018. Emerg Infect Dis. 2019;25:980–3. 10.3201/eid2505.190076 30848724 PMC6478227

[39] Vaughan A Aarons E Astbury J Brooks T Chand M Flegg P Human-to-Human Transmission of Monkeypox Virus, United Kingdom, October 2018. Emerg Infect Dis. 2020;26:782–5. 10.3201/eid2604.191164 32023204 PMC7101111

[40] Minhaj FS Ogale YP Whitehill F Schultz J Foote M Davidson W Monkeypox outbreak – Nine states, May 2022. MMWR Morb Mortal Wkly Rep. 2022;71:764–9. 10.15585/mmwr.mm7123e1 35679181 PMC9181052

[41] World Health Organization Multi-country monkeypox outbreak: situation update. 2022. Available: https://www.who.int/emergencies/disease-outbreak-news/item/2022-DON396. Accessed: 26 February 2025.

[42] Brown K Leggat PA Human monkeypox: current state of knowledge and implications for the future. Trop Med Infect Dis. 2016;1:8. 10.3390/tropicalmed1010008 30270859 PMC6082047

[43] Simpson K Heymann D Brown CS Edmunds WJ Elsgaard J Fine P Human monkeypox–After 40 years, an unintended consequence of smallpox eradication. Vaccine. 2020;38:5077–81. 10.1016/j.vaccine.2020.04.062 32417140 PMC9533855

[44] Patel VM Patel SV Epidemiological Review on Monkeypox. Cureus. 2023;15:e34653. 36895541 10.7759/cureus.34653 PMC9991112

[45] Pieretti VM Agriello M Delgado Molina MM Bonaura P Ramallo CA Miraglia E [Monkeypox: case series]. Rev Fac Cien Med Univ Nac Cordoba. 2023;80:321–34. Spanish. 10.31053/1853.0605.v80.n4.42303 38150198 PMC10851401

[46] Cassir N Cardona F Tissot-Dupont H Bruel C Doudier B Lahouel S Observational Cohort Study of Evolving Epidemiologic, Clinical, and Virologic Features of Monkeypox in Southern France. Emerg Infect Dis. 2022;28:2409–15. 10.3201/eid2812.221440 36241422 PMC9707593

[47] Tan DHS Pico Espinosa O Matelski J Khera SS Qamar A Persaud R Longitudinal Analysis of Mpox Virus DNA Detectability From Multiple Specimen Types During Acute Illness: A Cohort Study. Open Forum Infect Dis. 2024;11:ofae073. 10.1093/ofid/ofae073 38390463 PMC10883290

[48] Isidro J Borges V Pinto M Sobral D Santos JD Nunes A Phylogenomic characterization and signs of microevolution in the 2022 multi-country outbreak of monkeypox virus. Nat Med. 2022;28:1569–72. 10.1038/s41591-022-01907-y 35750157 PMC9388373

[49] Angelo KM Smith T Camprubí-Ferrer D Balerdi-Sarasola L Menéndez MD Servera-Negre G Epidemiological and clinical characteristics of patients with monkeypox in the GeoSentinel Network: a cross-sectional study. Lancet Infect Dis. 2023;23:196–206. 10.1016/S1473-3099(22)00651-X 36216018 PMC9546520

[50] Hammarlund E Lewis MW Carter SV Amanna I Hansen SG Strelow LI Multiple diagnostic techniques identify previously vaccinated individuals with protective immunity against monkeypox. Nat Med. 2005;11:1005–11. 10.1038/nm1273 16086024

[51] Lum FM Torres-Ruesta A Tay MZ Lin RTP Lye DC Rénia L Monkeypox: disease epidemiology, host immunity and clinical interventions. Nat Rev Immunol. 2022;22:597–613. 10.1038/s41577-022-00775-4 36064780 PMC9443635

[52] Huhn GD Bauer AM Yorita K Graham MB Sejvar J Likos A Clinical characteristics of human monkeypox, and risk factors for severe disease. Clin Infect Dis. 2005;41:1742–51. 10.1086/498115 16288398

[53] Adler H Gould S Hine P Snell LB Wong W Houlihan CF NHS England High Consequence Infectious Diseases (Airborne) Network. Clinical features and management of human monkeypox: a retrospective observational study in the UK. Lancet Infect Dis. 2022;22:1153–62. 10.1016/S1473-3099(22)00228-6 35623380 PMC9300470

[54] Hobson G Adamson J Adler H Firth R Gould S Houlihan C Family cluster of three cases of monkeypox imported from Nigeria to the United Kingdom, May 2021. Euro Surveill. 2021;26:2100745. 10.2807/1560-7917.ES.2021.26.32.2100745 34387184 PMC8365177

[55] O’Neil MJ Archer R Danza P Fisher R Bagwell DA Younis I Successful Distribution of Tecovirimat During the Peak of the Mpox Outbreak - Los Angeles County, June 2022-January 2023. MMWR Morb Mortal Wkly Rep. 2024;73:546–50. 38900699 10.15585/mmwr,mm7324a2 PMC11199022

[56] De Baetselier I Van Dijck C Kenyon C Coppens J Michiels J de Block T Retrospective detection of asymptomatic monkeypox virus infections among male sexual health clinic attendees in Belgium. Nat Med. 2022;28:2288–92. 10.1038/s41591-022-02004-w 35961373 PMC9671802

[57] Ahmed SK Mohamed MG Dabou EA Abuijlan I Chandran D El-Shall NA Monkeypox (mpox) in immunosuppressed patients. F1000 Res. 2023;12:127. 10.12688/f1000research.130272.2 37089133 PMC10113800

[58] Cassir N Cardona F Tissot-Dupont H Bruel C Doudier B Lahouel S Observational Cohort Study of Evolving Epidemiologic, Clinical, and Virologic Features of Monkeypox in Southern France. Emerg Infect Dis. 2022;28:2409–15. 10.3201/eid2812.221440 36241422 PMC9707593

[59] Ellingson MK Omer SB Sevdalis N Thomson A Validation of the Vaccination Trust Indicator (VTI) in a multi-country survey of adult vaccination attitudes. PLOS Glob Public Health. 2023;3:e0001820. 10.1371/journal.pgph.0001820 37043441 PMC10096184

[60] Sah R Siddiq A Mohanty A Rais MA Al-Ahdal T Padhi BK Monkeypox in children and elderly: correspondence. Ann Med Surg (Lond). 2023;85:550–2. 36923776 10.1097/MS9.0000000000000091 PMC10010777

[61] Benites-Zapata VA Ulloque-Badaracco JR Alarcon-Braga EA Hernandez-Bustamante EA Mosquera-Rojas MD Bonilla-Aldana DK Clinical features, hospitalisation and deaths associated with monkeypox: a systematic review and meta-analysis. Ann Clin Microbiol Antimicrob. 2022;21:36. 10.1186/s12941-022-00527-1 35948973 PMC9364300

[62] Adigun OA Okesanya OJ Ahmed MM Ukoaka BM Lucero-Prisno DE III Onyeaghala EO Syndemic Challenges: Addressing the Resurgence of Mpox Amidst Concurrent Outbreaks in the DRC. Transbound Emerg Dis. 2024;2024:1962224. 10.1155/tbed/1962224 PMC12020385 40303062

[63] Liu H Wang W Zhang Y Wang F Duan J Huang T Global perspectives on smallpox vaccine against monkeypox: a comprehensive meta-analysis and systematic review of effectiveness, protection, safety and cross-immunogenicity. Emerging Microbes &amp. Emerg Microbes Infect. 2024;13:2387442. 10.1080/22221751.2024.2387442 39082272 PMC11332295

[64] Fahrni ML Priyanka, Sharma A, Choudhary OP. Monkeypox: Prioritizing public health through early intervention and treatment. Int J Surg. 2022;104:106774. 10.1016/j.ijsu.2022.106774 35863624 PMC9293389

[65] Gruber MF Current status of monkeypox vaccines. NPJ Vaccines. 2022;7:94. 10.1038/s41541-022-00527-4 35977979 PMC9385639

[66] The Democratic Republic of the Congo Ministry of Public Health, Hygiene, and Prevention. Daily report of the Mpox epidemic in the DRC. 13 November 2024. Available: https://reliefweb.int/report/democratic-republic-congo/rapport-journalier-de-lepidemie-de-mpox-en-rdc-sitrep-ndeg85-donnees-du-13-novembre-2024-semaine-epidemiologique-46. Accessed: 3 February 2025.

[67] Whitehouse ER Bonwitt J Hughes CM Lushima RS Likafi T Nguete B Clinical and Epidemiological Findings from Enhanced Monkeypox Surveillance in Tshuapa Province, Democratic Republic of the Congo During 2011-2015. J Infect Dis. 2021;223:1870–8. 10.1093/infdis/jiab133 33728469

[68] Jiang RM Zheng YJ Zhou L Feng LZ Ma L Xu BP Diagnosis, treatment, and prevention of monkeypox in children: an experts’ consensus statement. World J Pediatr. 2023;19:231–42. 10.1007/s12519-022-00624-3 36409451 PMC9685019

[69] Chavda VP Apostolopoulos V Rare monkeypox: Is it really a threat to the elderly? Maturitas. 2022;163:90–1. 10.1016/j.maturitas.2022.05.014 35710608 PMC9192136

[70] Khanna U Bishnoi A Singh K Vinay K Clinical considerations in pediatric cases of monkeypox. J Am Acad Dermatol. 2023;88:e91–2. 10.1016/j.jaad.2022.09.009 36113613 PMC9677527

[71] Kumar N Acharya A Gendelman HE Byrareddy SN The 2022 outbreak and the pathobiology of the monkeypox virus. J Autoimmun. 2022;131:102855. 10.1016/j.jaut.2022.102855 35760647 PMC9534147

[72] Billioux BJ Mbaya OT Sejvar J Nath A Potential complications of monkeypox. Lancet Neurol. 2022;21:872. 10.1016/S1474-4422(22)00340-4 36115356 PMC9628741

[73] Petersen E Kantele A Koopmans M Asogun D Yinka-Ogunleye A Ihekweazu C Human monkeypox: epidemiologic and clinical characteristics, diagnosis, and prevention. Infectious Disease Clinics. 2019;33:1027–43. 30981594 10.1016/j.idc.2019.03.001 PMC9533922

[74] Lulli LG Baldassarre A Mucci N Arcangeli G Prevention, Risk Exposure, and Knowledge of Monkeypox in Occupational Settings: A Scoping Review. Trop Med Infect Dis. 2022;7:276. 10.3390/tropicalmed7100276 36288017 PMC9608671

[75] Vo C Zomorodi R Silvera R Bartram L Lugo LA Kojic E Clinical Characteristics and Outcomes of Patients With Mpox Who Received Tecovirimat in a New York City Health System. Open Forum Infect Dis. 2023;10:ofad552. 10.1093/ofid/ofad552 38023539 PMC10644828

[76] Reynolds MG Yorita KL Kuehnert MJ Davidson WB Huhn GD Holman RC Clinical manifestations of human monkeypox influenced by route of infection. J Infect Dis. 2006;194:773–80. 10.1086/505880 16941343

[77] Winters M Malik AA Omer SB Attitudes towards Monkeypox vaccination and predictors of vaccination intentions among the US general public. PLoS One. 2022;17:e0278622. 10.1371/journal.pone.0278622 36454991 PMC9714903

[78] Ndugga N, Haldar S, Pillai D, Hill L, Artiga S. Monkeypox (MPX) Cases and Vaccinations by Race/Ethnicity. Kaiser Family Foundation. 24 August 2022. Available: https://www.kff.org/racial-equity-and-health-policy/issue-brief/monkeypox-mpx-cases-and-vaccinations-by-race-ethnicity/. Accessed: 3 February 2025.

[79] Payne AB Incidence of monkeypox among unvaccinated persons compared with persons receiving≥ 1 JYNNEOS vaccine dose—32 US jurisdictions, July 31–September 3, 2022. MMWR Morb Mortal Wkly Rep. 2022;71:1278–82. 10.15585/mmwr.mm7140e3 36201401 PMC9541026

[80] Huhn GD Bauer AM Yorita K Graham MB Sejvar J Likos A Clinical Characteristics of Human Monkeypox, and Risk Factors for Severe Disease. Clin Infect Dis. 2005;41:1742–51. 10.1086/498115 16288398

[81] Mukinda VB Mwema G Kilundu M Heymann DL Khan AS Esposito JJ Re-emergence of human monkeypox in Zaire in 1996. Lancet. 1997;349:1449–50. 10.1016/S0140-6736(05)63725-7 9164323 PMC9533927

[82] Arita I Jezek Z Khodakevich L Ruti K Human monkeypox: a newly emerged orthopoxvirus zoonosis in the tropical rain forests of Africa. Am J Trop Med Hyg. 1985;34:781–9. 10.4269/ajtmh.1985.34.781 2992305

[83] Reed KD Melski JW Graham MB Regnery RL Sotir MJ Wegner MV The detection of monkeypox in humans in the Western Hemisphere. N Engl J Med. 2004;350:342–50. 10.1056/NEJMoa032299 14736926

[84] Alakunle EF Okeke MI Monkeypox virus: a neglected zoonotic pathogen spreads globally. Nat Rev Microbiol. 2022;20:507–8. 10.1038/s41579-022-00776-z 35859005 PMC9297671

[85] Slomski A Monkeypox Neurologic Complications May Be Similar to Smallpox. JAMA. 2022;328:1677. 10.1001/jama.2022.18441 36318144

[86] Dye C Kraemer MU Investigating the monkeypox outbreak. BMJ. 2022;377:o1314. 10.1136/bmj.o1314 35618293

[87] Perez Duque M Ribeiro S Martins JV Casaca P Leite PP Tavares M Ongoing monkeypox virus outbreak, Portugal, 29 April to 23 May 2022. Euro Surveill. 2022;27:2200424. 10.2807/1560-7917.ES.2022.27.22.2200424 35656830 PMC9164676

[88] Moore MJ, Rathish B, Zahra F. Monkeypox: Treasure Island, FL, USA: StatPearls Publishing; 2022. 34662033

[89] Weaver JR Isaacs SN Monkeypox virus and insights into its immunomodulatory proteins. Immunol Rev. 2008;225:96–113. 10.1111/j.1600-065X.2008.00691.x 18837778 PMC2567051

[90] Karagoz A Tombuloglu H Alsaeed M Tombuloglu G AlRubaish AA Mahmoud A Monkeypox (mpox) virus: Classification, origin, transmission, genome organization, antiviral drugs, and molecular diagnosis. J Infect Public Health. 2023;16:531–41. 10.1016/j.jiph.2023.02.003 36801633 PMC9908738

[91] Ghazy RM Elrewany E Gebreal A ElMakhzangy R Fadl N Elbanna EH Systematic Review on the Efficacy, Effectiveness, Safety, and Immunogenicity of Monkeypox Vaccine. Vaccines (Basel). 2023;11:1708. 10.3390/vaccines11111708 38006040 PMC10674429

[92] Chen N Li G Liszewski MK Atkinson JP Jahrling PB Feng Z Virulence differences between monkeypox virus isolates from West Africa and the Congo basin. Virology. 2005;340:46–63. 10.1016/j.virol.2005.05.030 16023693 PMC9534023

[93] Bunge EM Hoet B Chen L Lienert F Weidenthaler H Baer LR The changing epidemiology of human monkeypox-A potential threat? A systematic review. PLoS Negl Trop Dis. 2022;16:e0010141. 10.1371/journal.pntd.0010141 35148313 PMC8870502

[94] Al Awaidy ST Khamis F Sallam M Ghazy RM Zaraket H Monkeypox (mpox) Outbreak: More queries posed as cases soar globally. Sultan Qaboos Univ Med J. 2023;23:1–4. 10.18295/squmj.8.2022.046 36865422 PMC9974023

[95] O’Shea J Filardo TD Morris SB Weiser J Petersen B Brooks JT Interim Guidance for Prevention and Treatment of Monkeypox in Persons with HIV Infection - United States, August 2022. MMWR Morb Mortal Wkly Rep. 2022;71:1023–8. 10.15585/mmwr.mm7132e4 35951495 PMC9400540

[96] Nguyen PY Ajisegiri WS Costantino V Chughtai AA MacIntyre CR Reemergence of Human Monkeypox and Declining Population Immunity in the Context of Urbanization, Nigeria, 2017-2020. Emerg Infect Dis. 2021;27:1007–14. 10.3201/eid2704.203569 33756100 PMC8007331

[97] Ophinni Y Frediansyah A Sirinam S Megawati D Stoian AM Enitan SS Monkeypox: Immune response, vaccination and preventive efforts. Narra J. 2022;2:e90. 10.52225/narra.v2i3.90 38449905 PMC10914130

[98] Kenner J Cameron F Empig C Jobes DV Gurwith M LC16m8: an attenuated smallpox vaccine. Vaccine. 2006;24:7009–22. 10.1016/j.vaccine.2006.03.087 17052815 PMC7115618

[99] Hazra A Rusie L Hedberg T Schneider JA Human Monkeypox Virus Infection in the Immediate Period After Receiving Modified Vaccinia Ankara Vaccine. JAMA. 2022;328:2064–7. 10.1001/jama.2022.18320 36178700 PMC9526114

[100] Ghazy RM Yazbek S Gebreal A Hussein M Addai SA Mensah E Monkeypox Vaccine Acceptance among Ghanaians: A Call for Action. Vaccines (Basel). 2023;11:240. 10.3390/vaccines11020240 36851118 PMC9959510

[101] Karem KL Reynolds M Hughes C Braden Z Nigam P Crotty S Monkeypox-induced immunity and failure of childhood smallpox vaccination to provide complete protection. Clin Vaccine Immunol. 2007;14:1318–27. 10.1128/CVI.00148-07 17715329 PMC2168110

[102] Fine PE Jezek Z Grab B Dixon H The transmission potential of monkeypox virus in human populations. Int J Epidemiol. 1988;17:643–50. 10.1093/ije/17.3.643 2850277

[103] Rimoin AW Mulembakani PM Johnston SC Lloyd Smith JO Kisalu NK Kinkela TL Major increase in human monkeypox incidence 30 years after smallpox vaccination campaigns cease in the Democratic Republic of Congo. Proc Natl Acad Sci U S A. 2010;107:16262–7. 10.1073/pnas.1005769107 20805472 PMC2941342

[104] Priyamvada L Carson WC Ortega E Navarra T Tran S Smith TG Serological responses to the MVA-based JYNNEOS monkeypox vaccine in a cohort of participants from the Democratic Republic of Congo. Vaccine. 2022;40:7321–7. 10.1016/j.vaccine.2022.10.078 36344361 PMC9635871

[105] Wolff Sagy Y Zucker R Hammerman A Markovits H Arieh NG Abu Ahmad W Real-world effectiveness of a single dose of mpox vaccine in males. Nat Med. 2023;29:748–52. 10.1038/s41591-023-02229-3 36720271 PMC9930701

[106] van Ewijk CE Miura F van Rijckevorsel G de Vries HJ Welkers MR van den Berg OE Dutch Mpox Response Team; Members of the Dutch Mpox Response Team. Mpox outbreak in the Netherlands, 2022: public health response, characteristics of the first 1,000 cases and protection of the first-generation smallpox vaccine. Euro Surveill. 2023;28:2200772. 10.2807/1560-7917.ES.2023.28.12.2200772 36951783 PMC10037659

[107] Dukers-Muijrers NHTM Evers Y Widdershoven V Davidovich U Adam PCG Op de Coul ELM Mpox vaccination willingness, determinants, and communication needs in gay, bisexual, and other men who have sex with men, in the context of limited vaccine availability in the Netherlands (Dutch Mpox-survey). Front Public Health. 2023;10:1058807. 10.3389/fpubh.2022.1058807 36684959 PMC9850232

[108] Titanji BK Hazra A Zucker J Mpox Clinical Presentation, Diagnostic Approaches, and Treatment Strategies: A Review. JAMA. 2024;332:1652–62. 10.1001/jama.2024.21091 39401235

[109] Sun Y Nie W Tian D Ye Q Human monkeypox virus: Epidemiologic review and research progress in diagnosis and treatment. J Clin Virol. 2024;171:105662. 10.1016/j.jcv.2024.105662 38432097

[110] Khan G Perveen N Monkeypox: Past, Present, and Future. Adv Exp Med Biol. 2024;1451:1–20. 10.1007/978-3-031-57165-7_1 38801568

[111] Saalbach KP Treatment and Vaccination for Smallpox and Monkeypox. Adv Exp Med Biol. 2024;1451:301–16. 10.1007/978-3-031-57165-7_19 38801586

[112] Prévost J Sloan A Deschambault Y Tailor N Tierney K Azaransky K Treatment efficacy of cidofovir and brincidofovir against clade II Monkeypox virus isolates. Antiviral Res. 2024;231:105995. 10.1016/j.antiviral.2024.105995 39243894

[113] Mrosik S Rasokat H Fabri M Bopp L [Human monkeypox (Mpox)]. Dermatologie (Heidelb). 2024;75:40–7. German. 10.1007/s00105-023-05268-6 38063873

[114] Merad Y Gaymard A Cotte L Perpoint T Alfaiate D Godinot M Outcomes of post-exposure vaccination by modified vaccinia Ankara to prevent mpox (formerly monkeypox): a retrospective observational study in Lyon, France, June to August 2022. Euro Surveill. 2022;27:2200882. 10.2807/1560-7917.ES.2022.27.50.2200882 36695469 PMC9808316

[115] Ranganath N Tosh PK O’Horo J Sampathkumar P Binnicker MJ Shah AS Monkeypox 2022: Gearing Up for Another Potential Public Health Crisis. Mayo Clin Proc. 2022;97:1694–9. 10.1016/j.mayocp.2022.07.011 35985857

[116] Rao AK Schulte J Chen TH Hughes CM Davidson W Neff JM July 2021 Monkeypox Response Team. Monkeypox in a Traveler Returning from Nigeria - Dallas, Texas, July 2021. MMWR Morb Mortal Wkly Rep. 2022;71:509–16. 10.15585/mmwr.mm7114a1 35389974 PMC8989376

[117] Gong Q Wang C Chuai X Chiu S Monkeypox virus: a re-emergent threat to humans. Virol Sin. 2022;37:477–82. 10.1016/j.virs.2022.07.006 35820590 PMC9437600

[118] Elhusseiny SM Bebawy AS Saad BT Aboshanab KM Insights on monkeypox disease and its recent outbreak with evidence of nonsynonymous missense mutation. Future Sci OA. 2023;9:FSO877. 10.2144/fsoa-2023-0048 37485445 PMC10357398

[119] McCollum AM Nakazawa Y Ndongala GM Pukuta E Karhemere S Lushima RS Human Monkeypox in the Kivus, a Conflict Region of the Democratic Republic of the Congo. Am J Trop Med Hyg. 2015;93:718–21. 10.4269/ajtmh.15-0095 26283752 PMC4596588

[120] Li D Wilkins K McCollum AM Osadebe L Kabamba J Nguete Bet al. Evaluation of the GeneXpert for Human Monkeypox Diagnosis. Am J Trop Med Hyg. 2017;96:405–10. . 10.4269/ajtmh.16-0567 27994107 PMC5303045

[121] Hughes CM Liu L Davidson WB Radford KW Wilkins K Monroe B A Tale of Two Viruses: Coinfections of Monkeypox and Varicella Zoster Virus in the Democratic Republic of Congo. Am J Trop Med Hyg. 2020;104:604–11. 10.4269/ajtmh.20-0589 33289470 PMC7866336

[122] Mbala PK Huggins JW Riu-Rovira T Ahuka SM Mulembakani P Rimoin AW Maternal and Fetal Outcomes Among Pregnant Women With Human Monkeypox Infection in the Democratic Republic of Congo. J Infect Dis. 2017;216:824–8. 10.1093/infdis/jix260 29029147

[123] Schwartz DA Mbala-Kingebeni P Patterson K Huggins JW Pittman PR Congenital Mpox Syndrome (Clade I) in Stillborn Fetus after Placental Infection and Intrauterine Transmission, Democratic Republic of the Congo, 2008. Emerg Infect Dis. 2023;29:2198–2022. 10.3201/eid2911.230606 37705112 PMC10617360

[124] Gilchuk I Gilchuk P Sapparapu G Lampley R Singh V Kose N Cross-Neutralizing and Protective Human Antibody Specificities to Poxvirus Infections. Cell. 2016;167:684–694.e9. 10.1016/j.cell.2016.09.049 27768891 PMC5093772

[125] Sanchez Clemente N Coles C Paixao ES Brickley EB Whittaker E Alfven T Paediatric, maternal, and congenital mpox: a systematic review and meta-analysis. Lancet Glob Health. 2024;12:e572–88. 10.1016/S2214-109X(23)00607-1 38401556 PMC11519316

[126] See KC Vaccination for Mpox (Monkeypox) Infection in Humans: From Basic Science to Real-World Effectiveness. Vaccines (Basel). 2024;12:1147. 10.3390/vaccines12101147 39460314 PMC11511175

[127] Nakhaie M Pirmoradi Z Bashash D Rukerd MRZ Charostad J Beyond skin deep: shedding light on the neuropsychiatric consequences of Monkeypox (Mpox). Acta Neurol Belg. 2024;124:1189–97. 10.1007/s13760-023-02361-4 37624565

[128] Ogoina D Damon I Nakoune E Clinical review of human mpox. Clin Microbiol Infect. 2023;29:1493–501. 10.1016/j.cmi.2023.09.004 37704017

[129] Cevik M Tomori O Mbala P Scagliarini A Petersen E Low N The 2023 - 2024 multi-source mpox outbreaks of Clade I MPXV in sub-Saharan Africa: Alarm bell for Africa and the World. IJID Reg. 2024;12:100397. 10.1016/j.ijregi.2024.100397 39140010 PMC11321303

[130] Djuicy DD Sadeuh-Mba SA Bilounga CN Yonga MG Tchatchueng-Mbougua JB Essima GD Concurrent Clade I and Clade II Monkeypox Virus Circulation, Cameroon, 1979-2022. Emerg Infect Dis. 2024;30:432–43. 10.3201/eid3003.230861 38325363 PMC10902553

[131] Schwartz DA High Rates of Miscarriage and Stillbirth among Pregnant Women with Clade I Mpox (Monkeypox) Are Confirmed during 2023-2024 DR Congo Outbreak in South Kivu Province. Viruses. 2024;16:1123. 10.3390/v16071123 39066285 PMC11281436

[132] Sadeuh-Mba SA Yonga MG Els M Batejat C Eyangoh S Caro V Monkeypox virus phylogenetic similarities between a human case detected in Cameroon in 2018 and the 2017-2018 outbreak in Nigeria. Infect Genet Evol. 2019;69:8–11. 10.1016/j.meegid.2019.01.006 30634001 PMC9533929

[133] Resman Rus K Zakotnik S Sagadin M Kolenc M Skubic L Knap N Review of virological methods for laboratory diagnosis and characterization of monkeypox virus (MPXV): lessons learned from the 2022 Mpox outbreak. Acta Dermatovenerol Alp Pannonica Adriat. 2024;33:23–35. 10.15570/actaapa.2024.1 38179904

[134] Yu PA Elmor R Muhammad K Yu YC Rao AK Tecovirimat Use under Expanded Access to Treat Mpox in the United States, 2022-2023. NEJM Evid. 2024;3:a2400189. 10.1056/EVIDoa2400189 39270215 PMC11421955

[135] Kevin K Sinto R Nainggolan L Pasaribu A Shakinah S Lie KC Exploring the Potential Treatment for Mpox. Acta Med Indones. 2024;56:400–8. 39463117

[136] Braddick M Singh KP Therapeutic agents for the treatment of human mpox. Curr Opin Infect Dis. 2024;37:518-25. 10.1097/QCO.0000000000001069 39382085

[137] Packer S Patrzylas P Merrick R Sawyer C McAuley A Crowe W Mpox in UK households: estimating secondary attack rates and factors associated with transmission, May–November 2022. Epidemiol Infect. 2024;152:e113. 10.1017/S0950268824000864 39355858 PMC11450552

[138] Nolen LD Osadebe L Katomba J Likofata J Mukadi D Monroe B Extended Human-to-Human Transmission during a Monkeypox Outbreak in the Democratic Republic of the Congo. Emerg Infect Dis. 2016;22:1014–21. 10.3201/eid2206.150579 27191380 PMC4880088

[139] Fine PE Jezek Z Grab B Dixon H The transmission potential of monkeypox virus in human populations. Int J Epidemiol. 1988;17:643–50. 10.1093/ije/17.3.643 2850277

[140] Karem KL Reynolds M Hughes C Braden Z Nigam P Crotty S Monkeypox-induced immunity and failure of childhood smallpox vaccination to provide complete protection. Clin Vaccine Immunol. 2007;14:1318–27. 10.1128/CVI.00148-07 17715329 PMC2168110

[141] Mauldin MR McCollum AM Nakazawa YJ Mandra A Whitehouse ER Davidson W Exportation of Monkeypox Virus From the African Continent. J Infect Dis. 2022;225:1367–76. 10.1093/infdis/jiaa559 32880628 PMC9016419

[142] Ullah M Li Y Munib K Zhang Z Epidemiology, host range, and associated risk factors of monkeypox: an emerging global public health threat. Front Microbiol. 2023;14:1160984. 10.3389/fmicb.2023.1160984 37213509 PMC10196482

[143] Mahase E Monkeypox: Gay and bisexual men with high exposure risk will be offered vaccine in England. BMJ. 2022;377:o1542. 10.1136/bmj.o1542 35732317

[144] Wieder-Feinsod A Zilberman T Erster O Kolasko GW Biber A Gophen R Overlooked monkeypox cases among men having sex with men during the 2022 outbreak - a retrospective study. Int J Infect Dis. 2023;128:58–60. 10.1016/j.ijid.2022.12.014 36529372 PMC9754764

[145] Spicknall IH Pollock ED Clay PA Oster AM Charniga K Masters N Modelling the Impact of Sexual Networks in the Transmission of Monkeypox virus Among Gay, Bisexual, and Other Men Who Have Sex with Men - United States, 2022. MMWR Morb Mortal Wkly Rep. 2022;71:1131–5. 10.15585/mmwr.mm7135e2 36048619 PMC9472773

[146] Delaney KP Sanchez T Hannah M Edwards OW Carpino T Agnew-Brune C Strategies Adopted by Gay, Bisexual, and Other Men Who Have Sex with Men to Prevent Monkeypox virus Transmission - United States, August 2022. MMWR Morb Mortal Wkly Rep. 2022;71:1126–30. 10.15585/mmwr.mm7135e1 36048582 PMC9472779

[147] Holloway IW Lessons for Community-Based Scale-Up of Monkeypox Vaccination From Previous Disease Outbreaks Among Gay, Bisexual, and Other Men Who Have Sex With Men in the United States. Am J Public Health. 2022;112:1572–5. 10.2105/AJPH.2022.307075 35981275 PMC9558182

[148] Endo A Murayama H Abbott S Ratnayake R Pearson CAB Edmunds WJ Heavy-tailed sexual contact networks and monkeypox epidemiology in the global outbreak, 2022. Science. 2022;378:90–4. 10.1126/science.add4507 36137054

[149] Henderson DA The eradication of smallpox–an overview of the past, present, and future. Vaccine. 2011;29 Suppl 4:D7–9. 10.1016/j.vaccine.2011.06.080 22188929

[150] Simpson K Heymann D Brown CS Edmunds WJ Elsgaard J Fine P Human monkeypox - After 40 years, an unintended consequence of smallpox eradication. Vaccine. 2020;38:5077–81. 10.1016/j.vaccine.2020.04.062 32417140 PMC9533855

[151] Chakraborty C Bhattacharya M Ranjan Sharma A Dhama K Monkeypox virus vaccine evolution and global preparedness for vaccination. Int Immunopharmacol. 2022;113:109346. 10.1016/j.intimp.2022.109346 36274490 PMC9582788

[152] Reina J Iglesias C Vaccines against monkeypox. Med Clin (Engl Ed). 2023;160:305–9. 37033199 10.1016/j.medcle.2023.01.005 PMC10037303

[153] Jhancy M Poxvirus Vaccines: Past, Present, and Future. Adv Exp Med Biol. 2024;1451:273–87. 10.1007/978-3-031-57165-7_17 38801584

[154] Saadh MJ Ghadimkhani T Soltani N Abbassioun A Daniel Cosme Pecho R Taha A Progress and prospects on vaccine development against monkeypox infection. Microb Pathog. 2023;180:106156. 10.1016/j.micpath.2023.106156 37201635 PMC10186953

[155] Bhattacharya S The World Health Organization and global smallpox eradication. J Epidemiol Community Health. 2008;62:909–12. 10.1136/jech.2006.055590 18791049 PMC2602749

[156] Grabenstein JD Hacker A Vaccines against mpox: MVA-BN and LC16m8. Expert Rev Vaccines. 2024;23:796–811. 10.1080/14760584.2024.2397006 39188013

[157] Cotter CA Ignacio MA Americo JL Earl PL Mucker EM Frey TR Mpox mRNA-1769 vaccine inhibits orthopoxvirus replication at intranasal, intrarectal, and cutaneous sites of inoculation. npj vaccines. 2024;9:256. 10.1016/j.tim.2011.12.001 39719481 PMC11668852

[158] Ladhani SN Dowell AC Jones S Hicks B Rowe C Begum J Early evaluation of the safety, reactogenicity, and immune response after a single dose of modified vaccinia Ankara-Bavaria Nordic vaccine against mpox in children: a national outbreak response. Lancet Infect Dis. 2023;23:1042–50. 10.1016/S1473-3099(23)00270-0 37336224

[159] Shulman ST Monkeypox Emergence and the Eradication of Smallpox: An Historical Review. J Pediatric Infect Dis Soc. 2023;12:73–5. 10.1093/jpids/piac120 36409569

[160] Reynolds MG Damon IK Outbreaks of human monkeypox after cessation of smallpox vaccination. Trends Microbiol. 2012;20:80–7. 10.1016/j.tim.2011.12.001 22239910

[161] Grabenstein JD Hacker A Vaccines against mpox: MVA-BN and LC16m8. Expert Rev Vaccines. 2024;23:796–811. 10.1080/14760584.2024.2397006 39188013

[162] World Health Organization. Global response to monkeypox outbreaks: Vaccination campaigns and coordination efforts. Geneva, Switzerland: World Health Organization; 2023. Available: https://www.who.int/publications/m/item/mpox-global-strategic-preparedness-and-response-plan. Accessed: 3 February 2025.

[163] Garcia-Atutxa I Mondragon-Teran P Huerta-Saquero A Villanueva-Flores F Advancements in monkeypox vaccines development: a critical review of emerging technologies. Front Immunol. 2024;15:1456060. 10.3389/fimmu.2024.1456060 39464881 PMC11502315

[164] Afrough B Dowall S Hewson R Emerging viruses and current strategies for vaccine intervention. Clin Exp Immunol. 2019;196:157–66. 10.1111/cei.13295 30993690 PMC6468171

[165] Xiong Z Xue L Li X Zhang Y Assessing vaccine strategies for mpox outbreak in New York City using an age-structure model. BMC Infect Dis. 2024;24:1078. 10.1186/s12879-024-09551-2 39350073 PMC11441002

[166] World Health Organization. Strategic framework for enhancing prevention and control of mpox 2024-2027. Geneva, Switzerland: World Health Organization; 2024. Available: https://iris.who.int/bitstream/handle/10665/376839/9789240092907-eng.pdf. Accessed: 3 February 2025.

[167] Henley SJ Dowling ND Ahmad FB Ellington TD Wu M Richardson LC COVID-19 and Other Underlying Causes of Cancer Deaths – United States, January 2018-July 2022. MMWR Morb Mortal Wkly Rep. 2022;71:1583–8. 10.15585/mmwr.mm7150a3 36520660 PMC9762902

[168] Doshi S Shin S Lapointe-Shaw L Fowler RA Fralick M Kwan JL Temporal Clustering of Critical Events on Medical Wards. JAMA Intern Med. 2023;183:924–32. 10.1001/jamainternmed.2023.2629 37428478 PMC10334292

[169] JAMA Network. Mpox (Monkeypox) 2025. Available: https://jamanetwork.com/collections/46420/mpox-monkeypox. Accessed: 3 February 2025.

[170] Abdullah K Hussain J Chan E Tingley K Ly V Weese JS A Review of Evidence Related to the Zoonotic Characteristics of the Monkeypox Virus. Open Forum Infect Dis. 2024;11:S146–55. 39415826 10.1093/ofid/ofae503 PMC11476936

