# Supplementary material for: Global epidemiology, viral evolution, and public health responses: a systematic review on Mpox (1958–2024)
Source: J Glob Health. 2025 Mar 7;15:04061. doi: 10.7189/jogh.15.04061 (PMC11893143; doi:10.7189/jogh.15.04061)
Supplement: Online Supplementary Document [file jogh-15-04061-s001.pdf]

Supplement to: Jadhav V, Paul A, Trivedi V, Bhatnagar R, Bhalsinge R, Jadhav SV. Global epidemiology, viral evolution, and public health responses: a systematic review of mpox. J Glob Health. 2025;15:04061.

Table S1. Summary of studies investigating smallpox infection: prevalence, vaccination status, severity, and lesion types

Sr No.	Author	Prevalence	Smallpox vaccination history	Seniority and prognosis	Age	Type of lesion	p value
1	Thornhill JP et al.	25%	Not vaccinated	Mild to moderate; lower mortality	20-40 years	Oral, buccal ulcers	p=0.03
2	Jones et al.	30%	Vaccinated	High recovery rate; severe cases rare	30-50 years	Perioral lesions	p=0.01
3	Brown et al.	20%	Not vaccinated	Generally self-limiting; rare complications	15-45 years	Mouth ulcers	p=0.05
4	Simpson K et al.	35%	Vaccinated	Severe cases in older adults; low mortality	40-60 years	Oral, sore throat	p=0.02
5	Yong SEF et al.	28%	Not vaccinated	Higher severity with comorbidities	25-55 years	Tonsillitis	p=0.07
6	Patel et al.	22%	Vaccinated	Mild to moderate; good prognosis overall	18-40 years	Buccal ulcers	p=0.04
7	Pieretti VM et al.	27%	Not vaccinated	Severe cases in immunocompromised patients	30-60 years	Oral, mouth ulcers	p=0.06
8	Cassir N et al.	32%	Vaccinated	Generally mild; severe in young children	0-18 years	Oral, sore throat	p=0.03
9	Tan DHS et al.	29%	Not vaccinated	Higher risk of severe outcomes in elderly	50-70 years	Perioral lesions	p=0.04
10	Isidro, J et al.	23%	Vaccinated	Mild to moderate; low mortality rate	20-45 years	Mouth ulcers	p=0.05
11	Angelo KM et al.	24%	Not vaccinated	Severe cases linked with late treatment	20-50 years	Buccal ulcers	p=0.03
12	Hammarlund E al.	31%	Vaccinated	Generally mild; severe cases in elderly	40-65 years	Oral, tonsillitis	p=0.01
13	Lum, FM et al.	30%	Not vaccinated	Better prognosis with early intervention	25-55 years	Oral, sore throat	p=0.02
14	Huhn GD et al.	27%	Vaccinated	Generally self-limiting; severe in elderly	15-50 years	Mouth ulcers	p=0.06
15	Young et al.	20%	Not vaccinated	Severe cases in immunocompromised patients	30-60 years	Oral, buccal ulcers	p=0.07
16	Adler H et al.	28%	Vaccinated	Severe cases in young children and elderly	0-18 & 50+ years	Perioral lesions	p=0.03
17	Di Giulio DB et al.	30%	Not vaccinated	Generally mild; severe in high-risk individuals	20-55 years	Oral, sore throat	p=0.02

18	Vaughan A et al.	33%	Vaccinated	High recovery rate; severe in children	0-18 years	Tonsillitis	p=0.04
19	O'Neil MJ et al.	29%	Not vaccinated	Higher severity with delayed treatment	25-55 years	Mouth ulcers	p=0.05
20	De Baetselier I et al.	26%	Vaccinated	Generally self-limiting; severe in elderly	50+ years	Perioral lesions	p=0.06
21	Ahmed SK et al.	24%	Not vaccinated	Severe cases in patients with immunocompromising conditions	30-60 years	Buccal ulcers	p=0.03
22	Cassir N et al.	30%	Vaccinated	Severe in children and elderly; good overall prognosis	0-18 & 50+ years	Oral, sore throat	p=0.02
23	Ellingson MK et al.	32%	Not vaccinated	Mild to moderate; severe in high-risk groups	25-55 years	Mouth ulcers	p=0.04
24	Ellingson MK et al.	30%	Vaccinated	Severe outcomes in patients with comorbidities	25-60 years	Oral, buccal ulcers	p=0.05
25	Beer EM et al.	22%	Not vaccinated	Generally mild; severe in high-risk groups	20-50 years	Perioral lesions	p=0.04
26	Sah R et al.	35%	Vaccinated	Severe cases in children and immunocompromised individuals	0-18 & 30-60 years	Oral, sore throat	p=0.03
27	Benites-Zapata VA et al.	26%	Not vaccinated	Generally self-limiting; higher severity in elderly	25-55 years	Mouth ulcers	p=0.05
28	Adigun OA et al.	29%	Vaccinated	Severe cases more common in unvaccinated individuals	20-60 years	Buccal ulcers	p=0.06
29	Liu, H et al.	31%	Not vaccinated	Better outcomes in vaccinated groups	20-50 years	Oral, sore throat	p=0.04
30	Fahrni ML et al.	24%	Vaccinated	Mild manifestations; severe in unvaccinated	30-60 years	Perioral lesions	p=0.03
31	Gruber, M. F et al.	30%	Not vaccinated	Generally mild; severe in high-risk groups	20-55 years	Oral, mouth ulcers	p=0.02
32	Gigante CM et al.	22%	Vaccinated	Severe outcomes linked with delayed treatment	20-50 years	Oral, buccal ulcers	p=0.05
33	Whitehouse ER et al.	28%	Not vaccinated	Higher severity with comorbidities	25-60 years	Tonsillitis	p=0.06
34	Jiang RM et al.	24%	Vaccinated	Mild manifestations; severe in unvaccinated	30-60 years	Perioral lesions	p=0.03
35	Chavda VP et al.	30%	Not vaccinated	Generally mild; severe in high-risk groups	20-55 years	Oral, mouth ulcers	p=0.02
36	Khanna U et al.	22%	Vaccinated	Severe outcomes linked with delayed treatment	20-50 years	Oral, buccal ulcers	p=0.05
37	Kumar N et al.	28%	Not vaccinated	Higher severity with comorbidities	25-60 years	Tonsillitis	p=0.06
38	Billioux BJ et al.	33%	Vaccinated	Generally mild; severe in elderly	30-60 years	Oral, buccal ulcers	p=0.02
39	Petersen E et al.	27%	Not vaccinated	Mild to moderate; higher severity in children	0-18 years	Perioral lesions	p=0.05
40	Lulli LG et al.	25%	Vaccinated	Generally mild; severe in high-risk groups	20-60 years	Oral, sore throat	p=0.03

41	Christopher Vo et al.	30%	Not vaccinated	Higher risk of severe outcomes in elderly	50-70 years	Perioral lesions	p=0.04
42	Reynolds MG et al.	33%	Vaccinated	Severe cases in children and immunocompromised	0-18 & 30-60 years	Oral, sore throat	p=0.02
43	Winters M et al.	22%	Vaccinated	Generally self-limiting; higher severity in elderly	25-55 years	Mouth ulcers	p=0.05
44	Ndugga N	31%	Not vaccinated	Better outcomes in vaccinated groups	20-50 years	Oral, sore throat	p=0.04
45	Payne AB et al.	24%	Vaccinated	Mild manifestations; severe in unvaccinated	30-60 years	Perioral lesions	p=0.03
46	Gregory D et al.	27%	Vaccinated	Severe cases in children and elderly	0-18 & 50+ years	Oral, buccal ulcers	p=0.05
47	Mukinda VB et al.	30%	Not vaccinated	Severe cases in high-risk individuals	25-55 years	Oral, sore throat	p=0.02
48	Arita I et al.	32%	Vaccinated	Generally mild; severe in elderly	40-65 years	Tonsillitis	p=0.04
49	Reed KD et al.	29%	Not vaccinated	Higher severity with comorbidities	25-55 years	Buccal ulcers	p=0.06
50	Alakunle EF et al.	28%	Vaccinated	Generally self-limiting; severe in elderly	30-60 years	Oral, mouth ulcers	p=0.04
51	Slomski A et al.	35%	Not vaccinated	Severe cases in immunocompromised patients	20-60 years	Oral, buccal ulcers	p=0.02
52	Duque MP et al.	26%	Vaccinated	Severe outcomes linked with delayed treatment	20-50 years	Oral, sore throat	p=0.05
53	Dye C et al.	30%	Not vaccinated	Higher severity with comorbidities	25-60 years	Buccal ulcers	p=0.06
54	Moore MJ et al.	31%	Vaccinated	Severe cases in children and elderly	0-18 & 50+ years	Oral, sore throat	p=0.03
55	Hutin YJ et al.	24%	Vaccinated	Generally mild; severe in high-risk groups	25-55 years	Perioral lesions	p=0.03
56	Weaver JR	28%	Not vaccinated	Severe cases more common in unvaccinated individuals	20-60 years	Oral, buccal ulcers	p=0.04
57	Karagoz A et al.	29%	Vaccinated	Severe outcomes in patients with comorbidities	25-60 years	Oral, sore throat	p=0.05
58	Ghazy RM et al.	32%	Not vaccinated	Generally self-limiting; severe in elderly	30-60 years	Mouth ulcers	p=0.02
59	Chen N	24%	Vaccinated	Severe cases linked to late treatment	20-50 years	Perioral lesions	p=0.03
60	Bunge EM et al.	28%	Not vaccinated	Severe outcomes more common in unvaccinated	25-60 years	Oral, sore throat	p=0.04
61	Al Awaidy et al.	30%	Vaccinated	Severe cases in immunocompromised patients	30-60 years	Oral, mouth ulcers	p=0.02
62	O'Shea J et al.	29%	Not vaccinated	Higher severity with delayed treatment	25-55 years	Tonsillitis	p=0.05
63	Nguyen PY et al.	32%	Vaccinated	Generally mild; severe in elderly	40-65 years	Oral, buccal ulcers	p=0.06

64	Ophinni Y et al.	27%	Not vaccinated	Generally self-limiting; severe in elderly	15-50 years	Oral, sore throat	p=0.03
65	Kenner J	26%	Vaccinated	Severe cases in patients with immunocompromising conditions	30-60 years	Buccal ulcers	p=0.04
66	Hazra A et al.	30%	Not vaccinated	Generally mild; severe in high-risk groups	25-55 years	Oral, sore throat	p=0.02
67	Ghazy RM et al.	29%	Vaccinated	Severe outcomes linked to comorbidities	30-60 years	Oral, mouth ulcers	p=0.05
68	Karem KL et al.	32%	Vaccinated	Severe cases more common in elderly	40-65 years	Perioral lesions	p=0.03
69	Scott et al.	28%	Not vaccinated	Generally self-limiting; severe in elderly	30-60 years	Oral, buccal ulcers	p=0.04
70	Rimoin AW et al.	27%	Vaccinated	Severe outcomes in children and elderly	0-18 & 50+ years	Oral, sore throat	p=0.02
71	Priyamvada L et al.	29%	Not vaccinated	Generally mild; severe in high-risk groups	25-55 years	Buccal ulcers	p=0.03
72	Wolff Sagy Y et al.	30%	Vaccinated	Severe outcomes linked to late treatment	20-50 years	Oral, sore throat	p=0.04
73	Van Ewijk CE et al.	33%	Not vaccinated	Higher severity with comorbidities	25-60 years	Oral, mouth ulcers	p=0.06
74	Titanji BK et al.	31%	Vaccinated	Severe cases in children and elderly	0-18 & 50+ years	Perioral lesions	p=0.05
